# Supplementary material for: Immune-metabolic trajectories delineate subgroups in paediatric long COVID
Source: Nat Commun. 2026 May 4;17:4023. doi: 10.1038/s41467-026-72224-y (PMC13139442; doi:10.1038/s41467-026-72224-y)
Supplement: Supplementary file 1 — Supplementary Information [file 41467_2026_72224_MOESM1_ESM.pdf]

Immune-metabolic trajectories delineate subgroups in paediatric long COVID

Vilser et al. and Brunner-Weinzierl

Supplementary Figures and Tables

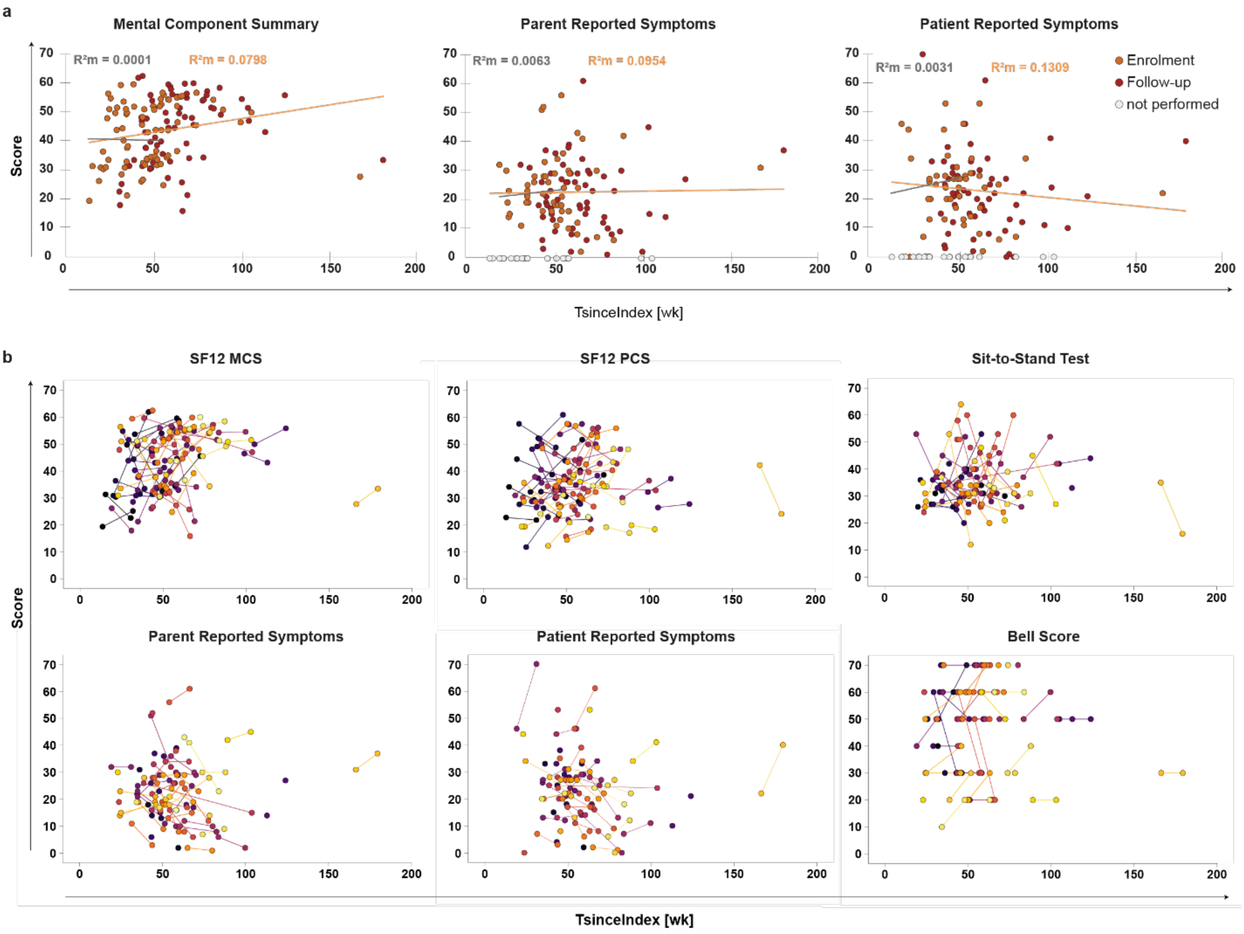

Supplementary Fig. 1 | Related to Fig. 1e. a, Additional LC-related scores versus TsinceIndex are shown for Mental Component Summary (n = 143 observations), parent-reported symptoms (n = 112 observations), and patient-reported symptoms (n = 104 observations). Associations were assessed using the same two-sided linear mixed-effects models (LMMs) as in Fig. 1e, with participant ID included as a random intercept. Numbers indicate marginal R^2 (R^2m) values for two time intervals: black numbers denote the first year post–SARS-CoV-2 infection, and red values denote the entire observational period. Each dot represents one assessment at one visit; most participants contributed data from two visits (see Supplementary Table 1b). Black and red lines are shown for visual guidance only and represent descriptive regression lines for the respective intervals. b, Selected symptom scores are plotted against TsinceIndex, with lines connecting repeated measurements from the same participant across visits. Shown are Mental Component Summary (SF-12 MCS; n = 143 observations), Physical Component Summary (SF-12 PCS; n = 143 observations), Sit-to-Stand test (n = 120 observations), parent-reported symptoms (n = 112 observations), patient-reported symptoms (n = 104 observations), and Bell score (n = 121 observations). Each dot represents one assessment at one visit.

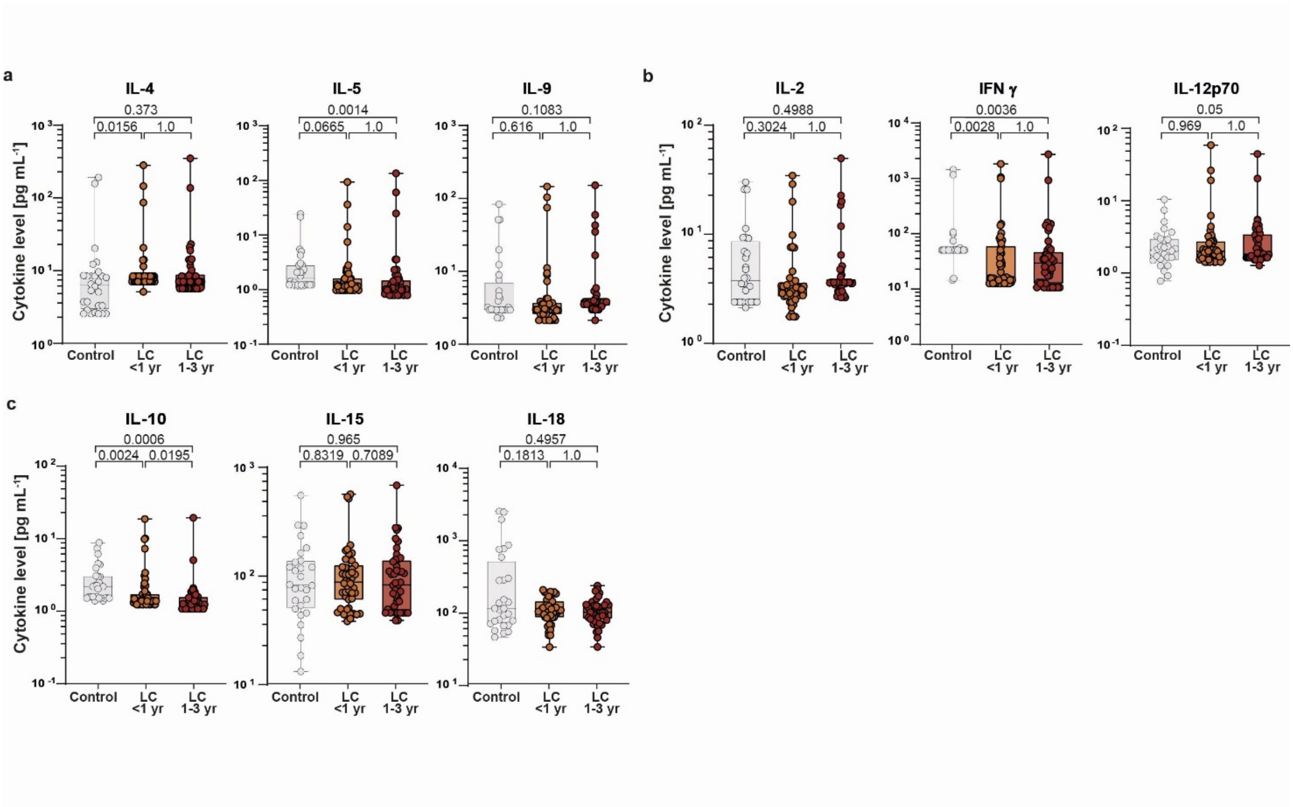

Supplementary Fig. 2 | Systemic cytokines not significantly elevated in paediatric LC. a-c, Cytokine concentrations in controls (n = 27) and paediatric LC stratified by TsinceIndex; repeated measurements were averaged within participant and TsinceIndex, yielding one value per participant per window (< 1 yr, n = 47; 1–3 yr, n = 45). Overall group differences were assessed using two-sided Kruskal–Wallis tests followed by unadjusted Dunn's post hoc pairwise comparisons versus controls. P values from Dunn's post hoc tests were adjusted using the Holm–Bonferroni method. Within-LC comparisons across TsinceIndex windows were assessed using two-sided linear mixed-effects models (LMMs), with TsinceIndex as a fixed effect and participant ID as a random intercept (see Supplementary Table 2.4). Cytokines shown here did not differ significantly from controls and did not increase significantly between LC < 1 yr and LC 1–3 yr. Box plots show the median (centre line) and interquartile range (box, 25th–75th percentiles); whiskers indicate the minimum and maximum values. Exact P values, as well as Holm–Bonferroni-adjusted P values, are reported in Supplementary Table 2.3.

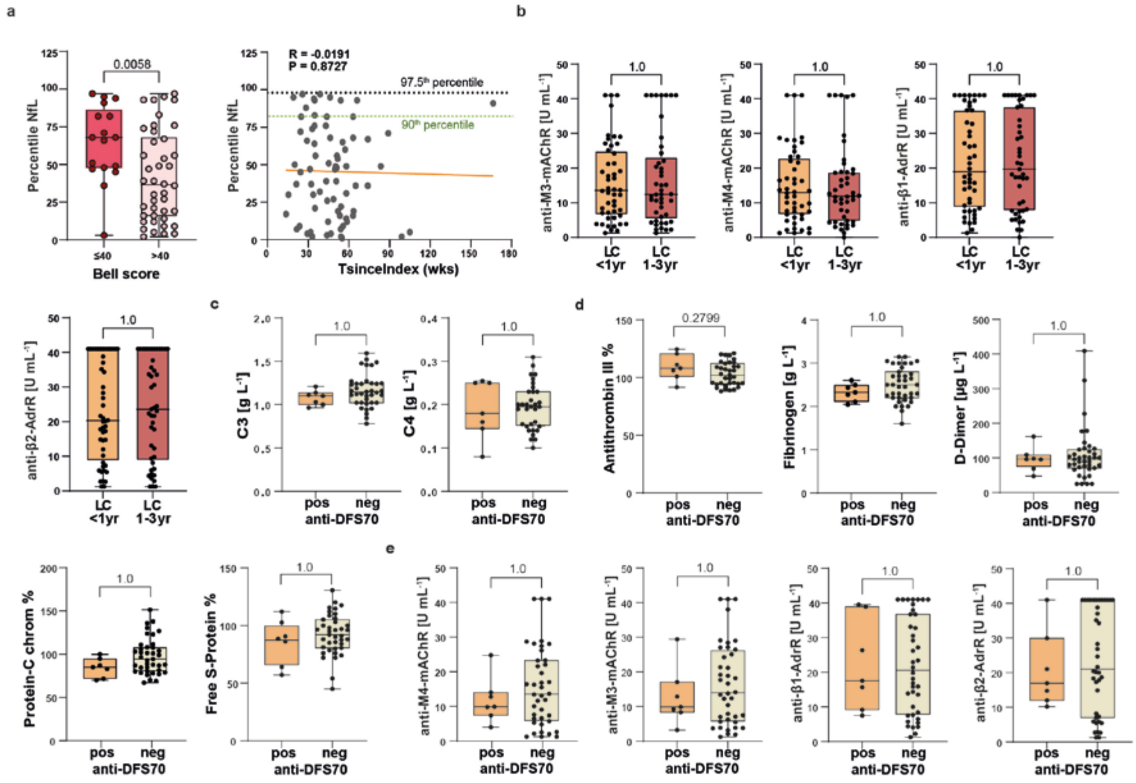

Supplementary Fig. 3 | NfL, autoantibodies, complement, coagulation and anti-DFS70. **a** Serum NfL percentile levels in LC patients at enrolment. **Left**, NfL percentiles stratified by Bell score (≤40, n = 17; red); >40, n = 42; pink). Statistical analysis was performed using a two-sided Welch's t-test (mean difference = −22.60, 95% CI = −38.22 to −6.99, P = 0.0058, effect size = 0.1969). **Right**, NfL percentiles (n = 73) were plotted against TsinceIndex (wk); Pearson's correlation coefficient r, two-sided P value and the fitted regression line (brown) are shown. The 97.5th and 90th percentiles are indicated as dotted reference lines. **b,** AAb readouts against muscarinic acetylcholine receptors (anti-M-mAChR) and β-adrenergic receptors (anti-β-AdrR) in paediatric LC stratified by TsinceIndex. For visualisation, LC patients were grouped in TsinceIndex windows and repeated measurements were averaged within each participant and TsinceIndex window to one value per individual (< 1 yr, n = 48; light orange; 1–3 yr, n = 45; dark orange). Within-LC comparisons over the complete time course were assessed using a two-sided LMM (n = 139 observations), with TsinceIndex as a fixed effect and participant ID as a random intercept; P values were adjusted for multiple comparisons using Holm–Bonferroni. **c**, Concentrations of complement factors C3 and C4 in anti-DFS70-positive patients (orange) versus anti-DFS70-negative patients (beige). P values were adjusted using the Holm–Bonferroni method. **d**, Box plots showing coagulation factors in anti-DFS70-positive (n = 7; orange) and anti-DFS70-negative patients (n = 39; beige), as indicated. **e**, AAb readouts against muscarinic acetylcholine receptors (anti-M-mAChR) and β-adrenergic receptors (anti-β-AdrR) in anti-DFS70-positive (orange) versus anti-DFS70-negative (beige) paediatric LC participants, as indicated. For **c–e**, associations were assessed using a two-sided LMM; participant ID included as a random intercept; P values were adjusted using the Holm–Bonferroni method. **a–e,** Each dot represents an individual participant; box plots show the median (centre line) and interquartile range (box, 25th–75th percentiles); whiskers indicate the minimum and maximum values. Exact P values are reported for b in Supplementary Table 3.2, for c–e in Supplementary Table 3.4.

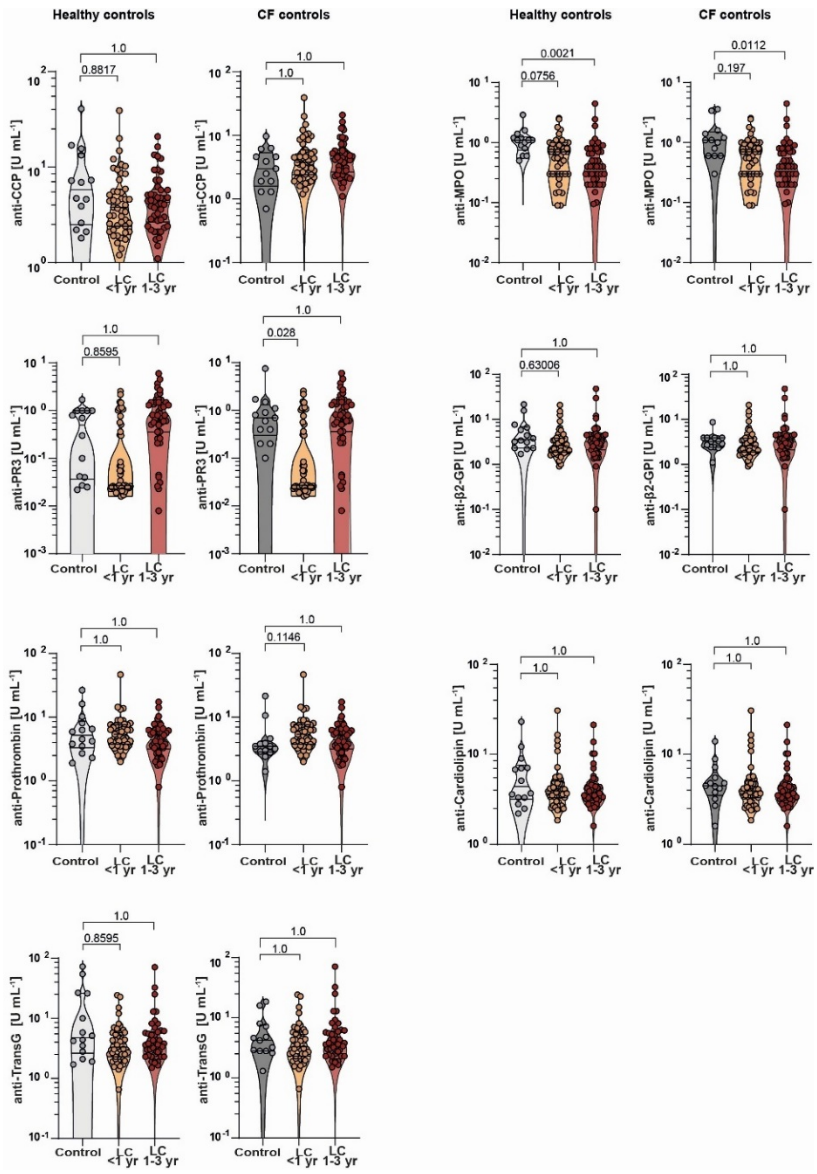

Supplementary Fig. 4 | Sensitivity analysis of pooled controls (Fig. 3): autoantibodies using stratified control subgroups. AAb titres (anti-CCP, anti-MPO, anti-PR3, anti–β2-glycoprotein I (anti-β2GPI), anti-Cardiolipin and anti-Prothrombin; U mL^{-1}; $\log_{10}$ scale) are shown. Overall group differences were assessed using a two-sided Kruskal–Wallis test, followed by adjusted Dunn's post hoc pairwise comparisons versus controls. Left panels: healthy controls (light grey) versus LC subgroups (LC < 1 yr light orange; 1–3 yr dark orange). Right panels: clinically stable cystic fibrosis (CF) controls (dark grey) versus LC subgroups (LC < 1 yr light orange; 1–3 yr dark orange). Violin plots show distributions with individual data points overlaid; centre line indicates the median and boxes the IQR; whiskers indicate the minimum and maximum values. P values are reported in Supplementary Table 3.3b, c in addition to adjusted P values using the Holm–Bonferroni method across all autoantibodies (n = 6). This figure serves as a sensitivity analysis for the pooled-control comparisons reported in Fig. 3. Exact P values are reported in Supplementary Table 3.3. The direction of effects was similar for both control subgroups and the pooled-control analysis shown in Figs. 2 and 3.

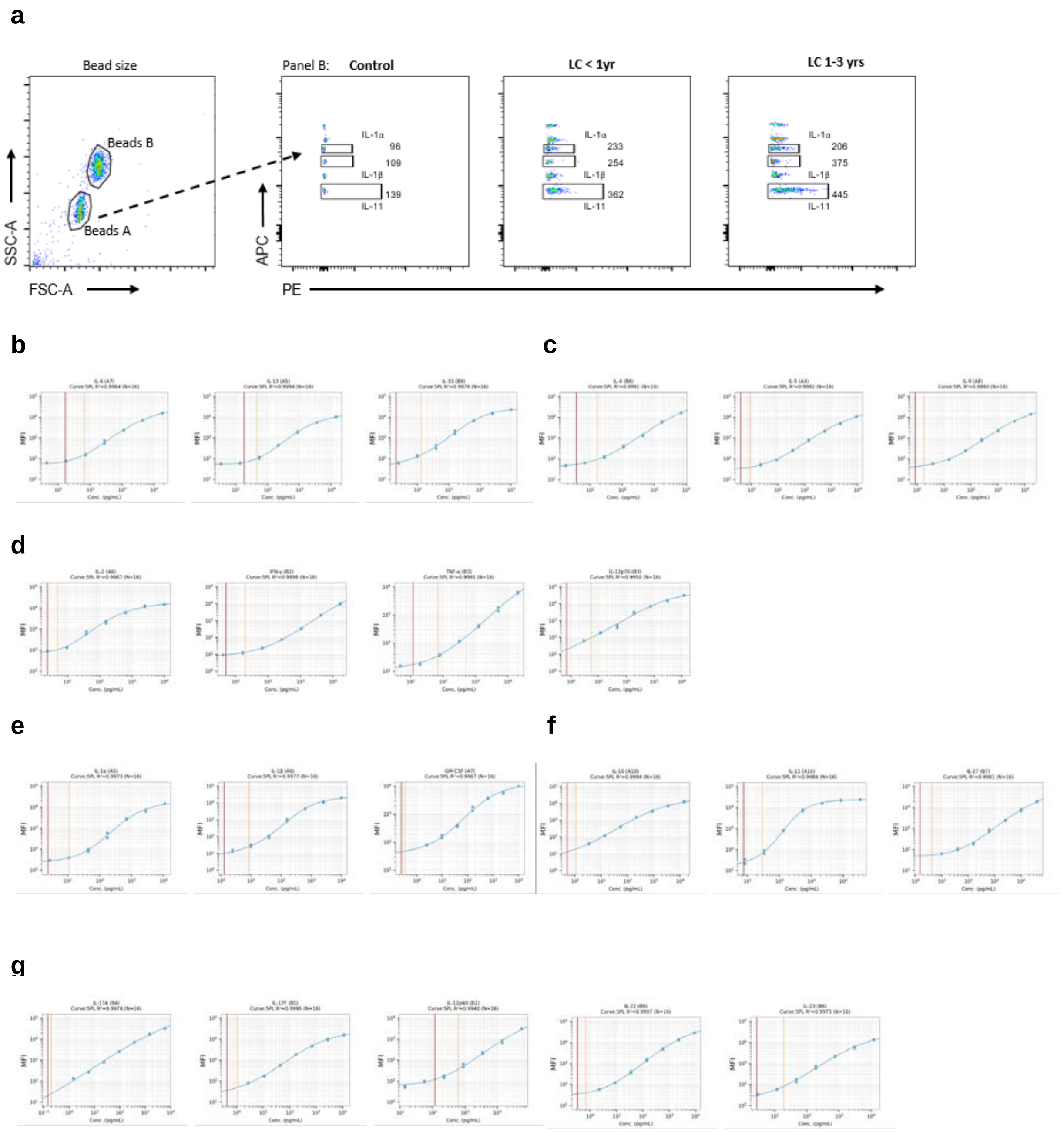

Supplementary Fig. 5 | Standard settings for cytokine quantification. (**a**) Representative dot plots of multiplex bead-based assay serum analysis from control group, LC patients within the first year and later years of disease. Analysis is shown for the Human Cytokine Panel 2 cytokines IL-1α, IL-1β, and IL-11 among others. Each population represents the acquisition of a specific cytokine, the numbers represent the mean fluorescence intensity (MFI). (**b-g**) Standard curve concentration range measured for cytokines in the LEGENDplex Human Th Cytokine Panel or LEGENDplex Human Cytokine Panel 2 for (**b**) SARS-CoV-2 related cytokines, (**c**) Th2/9 related cytokines, (**d**) Th1 related cytokines, (**e**) innate like cytokines, (**f**) regulatory cytokines and (**g**) Th17/22 related cytokines. The blue line represents the measured standard curve (mean fluorescence intensity), which was fitted using a five-parameter logistic (5PL) model with the LEGENDplex™ Data Analysis Software. The red vertical line indicates the calculated limit of detection (LOD) and the orange line indicates the limit of quantification (LOQ). These limits are derived from the fitted curve in the software and represent the theoretical concentrations at which the assay can detect and quantify the analyte, respectively.

Supplementary Table 1a | Demographic and clinical characteristics of participants. Values are shown as mean/SD or n (%), unless otherwise indicated. Group comparisons were performed using two-sided Pearson's χ^2 tests for categorical variables, and χ^2 statistics with corresponding P values are reported in the table unless otherwise indicated. Although age is displayed as a continuous variable (mean/SD), the reported group comparison for age is based on the prespecified age category of 0–14 years versus >14 years, as shown in Fig. 1b. Holm–Bonferroni-adjusted P values were applied only to the prespecified comparisons of age category and sex; all remaining P values are unadjusted. Atopy was defined based on parent-reported physician-diagnosed atopy, excluding drug allergies, and was analysed only among participants with known atopy status. VOC denotes variant of concern. In cases in which two SARS-CoV-2 variants qualified as variants of concern (VOCs), the later-emerging variant was assigned because of its higher transmissibility (Robert-Koch Institute (2025), Berlin, Germany. www.rki.de (Accessed: 11_03_2025). For participants with multiple infections, t_0 was defined as the infection after which LC symptoms first appeared, typically the most recent infection preceding LC onset.

	LC patients	Control participants	LC to Control χ^2 (P)
**General**			
N	74	27	
Age (mean/SD)	14.1/2.5	10.9/5.15	0.0589 (0.5800)
Sex female	43 (58 %)	15 (56 %)	1.1300 (0.6341)
Percentile BMI (mean/SD)	48.5/31.7		
Enrollment (TsinceIndex) (wk) (mean/SD)	47.5/23.2		
Follow-up (TsinceIndex) (wk) (mean/SD)	64.2/24.0		
Pre-born (< 37 GA) yes/no/not reported	7/66/1	0/0/27	
**SARS-CoV-2-related**			
Tested for COVID-19			
Anti-Nucleocapsid ab	72/74		
Anti-Spike ab	71/74		
Parents reported yes/no/not reported	74/0/0	10/10/7	
SARS-CoV-2 infections prior LC, Nº 1/2/3	45/28/1		
SARS-CoV-2 vaccinations prior LC, Nº 0/1/2/3/4	37/9/21/6/1		
**Chronic disease prior LC**			
Attention Deficit Hyperactivity Disorder (ADHD)	4 (5.4 %)		
Lactose or Fructose Malabsorption	7 (9.5 %)		
Obesity	2 (2.7 %)		
Dyslexia	2 (2.7 %)		
Migraine (including chronic headaches)	4 (5.4 %)		
Gilbert-Meulengracht-Syndrome	1 (1.4 %)		
Fetal Alcohol Spectrum Disorder	1 (1.4 %)		
Loeys-Dietz Syndrome	1 (1.4 %)		
Non-Alcoholic Fatty Liver Disease	1 (1.4%)		
Scoliosis	1 (1.4%)		

	LC patients	Control participants	LC to Control χ^2 (P)
Acne	1 (1.4 %)		
Visual Disturbances	1 (1.4 %)		
Sensory Processing Disorder	1 (1.4 %)		
Bronchial Asthma	3 (4.1 %)		
Coronary Fistula,<br>Tricuspid Functionally Bicuspid Aortic Valve,<br>Mild Mitral Regurgitation	1 (1.4%)		
Divergent Strabismus	1 (1.4 %)		
Myofascial Pain Syndrome (Thoracolumbar)	1 (1.4 %)		
Psychogenic Gait Disorder, Suspected Arterial Hypertension	1 (1.4 %)		
Hashimoto's Thyroiditis	1 (1.4 %)		
Chromosome 13 Anomaly	1 (1.4 %)		
Cystic Fibrosis	0 (0 %)	13 (48 %)	40.8800 (< 0.0001)
Atopy (excluding drug allergies) yes/no/unknown	26/48/0	3/16/8	1.6070 (0.2049)
No Prior Illness	37 (50.0 %)	14 (52 %)	0.0274 (0.8685)
**Dominant VOC in Germany during SARS-CoV-2 infection,** N^{o} (%)			
Ancestral and Alpha	6 (8.1 %)		
Delta	18 (24.3 %)		
Omicron	50 (67.6 %)		

* Group comparison for age is based on the prespecified age category of 0–14 years versus >14 years. Holm–Bonferroni-adjusted P values were applied to age category and sex (see Fig. 1b).

Supplementary Table 1b | Upper part: Lack of physical and mental health recovery in paediatric LC patients over time. **Lower part**: Summary details of two-sided LMMs for predisposing factors of paediatric LC and their association with disease severity within the first year after LC onset and across the whole disease course. Related to Fig. 1e. Dependent variables are listed in the leftmost column. In the upper part, two-sided LMMs included TsinceIndex as the only fixed effect and participant ID as a random intercept. In the lower part, extended two-sided LMMs included TsinceIndex, comorbidity, number of vaccinations, sex, and age as fixed effects, with participant ID included as a random intercept to account for repeated measurements. Marginal R^2 (R^2m), reflecting the variance explained by the fixed effects, and corresponding raw P values are shown for models within the first year after LC onset and across the whole disease course; Holm–Bonferroni-adjusted P values are additionally reported for the extended models. In whole-disease-course models, TsinceIndex was modelled as a continuous covariate. Additional lower panels summarize the corresponding model outputs for each outcome, reporting marginal R^2, F statistics with numerator and residual degrees of freedom, parameter estimates, 95% confidence intervals, and raw P values. Multiple-testing correction was performed using the Holm–Bonferroni method. Abbreviations: CSHQ, Children's SleeP Habits Questionnaire; FSS, Fatigue Severity Scale; GAD-7, Generalized Anxiety Disorder 7-item scale; KIDSCREEN, KIDSCREEN Questionnaire; PHQ-9, Patient Health Questionnaire 9-item; SF-12 MCS, Short Form-12 Mental Component Summary; SF-12 PCS, Short Form-12 Physical Component Summary; STS, Sit-to-Stand Test.

	LC < 1 yr		LC whole disease course			LC whole disease course Extended for potential confounders		
**Outcome**	R²m	P	R²m	P	Adj. P	R²m	P	Adj. P
SF-12 PCS	0.0077	0.4360	0.0387	0.0308	0.2980	0.1194	0.0936	0.8424
Bell score	0.0215	0.1498	0.0059	0.4176	1.0000	0.0969	0.3534	1.0000
SF-12 MCS	0.0001	0.9085	0.0150	0.1582	0.7910	0.0798	0.4276	1.0000
PHQ-9	0.0003	0.8574	0.0715	0.0017	0.0187	0.1046	0.0661	0.6610
GAD-7	0.0033	0.5359	0.0346	0.0298	0.2980	0.0595	0.5244	1.0000
FSS	0.0001	0.9154	0.0147	0.1703	0.7910	0.0445	0.7531	1.0000
Sit-to-Stand	0.0095	0.4619	0.0004	0.8365	1.0000	0.0350	0.8796	1.0000
KIDSCREEN-10	0.0007	0.7870	0.0332	0.0429	0.3430	0.0992	0.1904	1.0000
Parents-reported symptoms (acute)	0.0063	0.3962	0.0177	0.1312	0.7872	0.0954	0.3468	1.0000
Patients-reported symptoms (acute)	0.0031	0.6277	0.0070	0.3759	1.0000	0.1309	0.1976	1.0000
CSHQ Post	0.0163	0.1403	0.0306	0.0492	0.3432	0.1754	0.0086	0.0946

**Outcome**	obs (n)	R²m	F (df1, df2)	Estimate	95% CI
SF-12 PCS	71	0.0077	0.6152 (1, 57.9754)	0.9617	-1.6327 to 3.2216
Bell score	57	0.0215	2.2046 (1, 25.6833)	3.3658	-1.1809 to 7.9126
SF-12 MCS	71	0.0001	0.0134 (1, 36.9996)	0.1116	-1.8133 to 2.0365
PHQ-9	71	0.0003	0.0327 (1, 39.9166)	-0.0085	-0.1020 to 0.0838
GAD-7	71	0.0033	0.3900 (1, 39.9347)	-0.2625	-1.0393 to 0.5526
FSS	71	0.0001	0.0114 (1, 40.9838)	0.0144	-0.2324 to 0.2901
Sit-to-Stand	58	0.0095	0.5487 (1, 56.0000)	1.0707	-1.6073 to 3.6838
KIDSCREEN-10	71	0.0007	0.0739 (1, 44.2334)	-0.1983	-1.6648 to 1.2983
Parents-reported symptoms (acute)	49	0.0063	0.7625 (1, 15.0926)	-0.9424	-2.9751 to 1.2933
Patients-reported symptoms (acute)	46	0.0031	0.2427 (1, 19.8793)	-0.8713	-4.3426 to 2.7957
CSHQ Post	66	0.0163	2.2844 (1, 32.4731)	-0.9825	-2.3042 to 0.2920

**Outcome**	obs (n)	R²m	F (df1, df2)	Estimate	95% CI
SF-12 PCS	141	0.0387	4.7776 (1, 121.3861)	2.2258	0.2304 to 4.2190
Bell score	121	0.0059	0.6616 (1, 118.0879)	1.6777	-2.4072 to 5.7626
SF-12 MCS	141	0.015	2.0126 (1, 138.2573)	1.3858	-0.5458 to 3.3174
PHQ-9	141	0.0715	10.2400 (1, 138.4613)	-0.0588	-0.0934 to -0.0216
GAD-7	141	0.0346	4.8220 (1, 138.7378)	-0.8741	-1.6058 to -0.1301
FSS	139	0.0147	1.9005 (1, 135.7324)	-0.1892	-0.4487 to 0.0688
Sit-to-Stand	115	0.0004	0.0428 (1, 113.0000)	-0.1806	-1.8232 to 1.5667
KIDSCREEN-10	141	0.0332	4.1804 (1, 130.3642)	1.633	0.0178 to 3.0429
Parents-reported symptoms (acute)	112	0.0177	2.3145 (1, 103.0605)	-1.6274	-3.6211 to 0.5942
Patients-reported symptoms (acute)	104	0.007	0.7913 (1, 98.9570)	-1.1288	-3.4045 to 1.4820
CSHQ Post	134	0.0306	3.9399 (1, 131.4937)	-1.2849	-2.4790 to -0.0358

Lower part | Predisposing factors for paediatric LC do not associate with disease severity. Two-sided LMMs across the whole LC disease course were fitted with disease severity outcomes, including Bell score, questionnaire-based measures, and sit-to-stand performance. Fixed effects included comorbidity (yes vs no), sex (male = 1, female = 0), SARS-CoV-2 variant of concern (VOC) wave, number of infections prior to LC onset, number of vaccinations, TsinceIndex (wk), and age (yr); participant ID was modelled as a random intercept. Degrees of freedom were approximated using Satterthwaite's method, and 95% confidence intervals were obtained from 1,000 bootstrap samples. Shown are marginal R^2 values (R^2m), F statistics with numerator and residual degrees of freedom, fixed-effect estimates, 95% confidence intervals (CI), and raw P values; Holm–Bonferroni-adjusted P values are additionally reported for the extended models in Supplementary Table 1b, upper part. Positive estimates indicate higher outcome values relative to the reference category or per unit increase in the predictor. Continuous predictors were standardized. VOC wave was tested as a global omnibus factor; only omnibus F statistics are shown in this summary table. Individual category contrasts of each VOC wave are not displayed.

**SF12 PCS**				
**Predictor**	**F (df1, df2)**	**Estimate**	**95% CI**	**P**
Comorbidity	0.5600 (1, 65.3422)	-3.0869	-11.2485 to 5.0747	0.4570
Time since last infection (wk)	4.5990 (1, 130.9852)	2.9366	0.2273 to 5.6459	0.0338
Number of vaccinations	0.3351 (1, 67.4851)	0.7201	-1.7410 to 3.1812	0.5646
Age	2.1222 (1, 70.9744)	-1.7549	-4.1384 to 0.6285	0.1496
Sex (M=1, F=0)	1.9002 (1, 68.4230)	3.3895	-1.4754 to 8.2545	0.1725
Infections since LC onset	0.4874 (1, 66.4257)	0.8654	-1.5871 to 3.3179	0.4875
VOC wave	1.1936 (3, 69.8388)			0.3186

**Bell score**				
**Predictor**	**F (df1, df2)**	**Estimate**	**95% CI**	**P**
Comorbidity	1.2924 (1, 60.1293)	-10.1347	-27.8038 to 7.5343	0.2601
Time since last infection (wk)	0.9240 (1, 95.4803)	2.6750	-2.8403 to 8.1903	0.3388
Number of vaccinations	1.4056 (1, 67.9018)	-3.2270	-8.6217 to 2.1677	0.2399
Age	2.9240 (1, 73.6458)	4.4614	-0.7096 to 9.6324	0.0915
Sex (M=1, F=0)	1.8030 (1, 70.3705)	7.1735	-3.4149 to 17.7620	0.1837
Infections since LC onset	0.1607 (1, 66.6720)	-1.0839	-6.4430 to 4.2752	0.6898
VOC wave	0.7066 (3, 73.3764)			0.5511

**SF12 MCS**				
**Predictor**	**F (df1, df2)**	**Estimate**	**95% CI**	**P**
Comorbidity	0.0005 (1, 63.9367)	-0.0999	-9.2065 to 9.0068	0.9828
Time since last infection (wk)	2.5236 (1, 116.1654)	1.9484	-0.4783 to 4.3751	0.1149
Number of vaccinations	0.4374 (1, 69.7870)	-0.9082	-3.6252 to 1.8088	0.5106
Age	2.7907 (1, 76.4903)	2.1925	-0.4042 to 4.7892	0.0989
Sex (M=1, F=0)	0.1985 (1, 70.4260)	1.2064	-4.1511 to 6.5638	0.6573
Infections since LC onset	0.0655 (1, 67.3989)	0.3518	-2.3676 to 3.0712	0.7988
VOC wave	1.0488 (3, 70.1080)			0.3765

**PHQ-9**				
**Predictor**	**F (df1, df2)**	**Estimate**	**95% CI**	**P**
Comorbidity	0.2679 (1, 65.2099)	1.1118	-3.2105 to 5.1007	0.6065

Time since last infection (wk)	10.9641 (1, 117.8810)	-1.9200	-3.0522 to -0.8317	0.0012
Number of vaccinations	0.1949 (1, 70.8146)	-0.2831	-1.5601 to 0.9624	0.6602
Age	2.4997 (1, 77.3251)	0.9699	-0.1664 to 2.2829	0.1180
Sex (M=1, F=0)	0.0460 (1, 71.5129)	-0.2712	-2.7087 to 2.2526	0.8308
Infections since LC onset	0.4677 (1, 68.5373)	-0.4389	-1.7189 to 0.7897	0.4963
VOC wave	0.7534 (3, 71.3377)			0.5240

**GAD-7**				
**Predictor**	**F (df1, df2)**	**Estimate**	**95% CI**	**P**
Comorbidity	0.1469 (1, 64.0794)	0.7399	-2.8061 to 4.6602	0.7028
Time since last infection (wk)	3.9282 (1, 114.0392)	-0.9965	-2.0017 to -0.0747	0.0499
Number of vaccinations	1.3680 (1, 70.3584)	-0.6727	-1.8649 to 0.5133	0.2461
Age	1.2044 (1, 77.3295)	0.6023	-0.4794 to 1.7046	0.2759
Sex (M=1, F=0)	0.0050 (1, 70.8732)	-0.0799	-2.3701 to 2.1551	0.9440
Infections since LC onset	0.0909 (1, 67.7733)	-0.1737	-1.2652 to 0.9806	0.7639
VOC wave	0.3045 (3, 70.3033)			0.8221

**FSS**				
**Predictor**	**F (df1, df2)**	**Estimate**	**95% CI**	**P**
Comorbidity	0.1847 (1, 65.4013)	0.2794	-1.0527 to 1.5825	0.6688
Time since last infection (wk)	4.1631 (1, 114.9780)	-0.3574	-0.6951 to 0.0015	0.0436
Number of vaccinations	0.4699 (1, 71.7878)	0.1334	-0.2302 to 0.4988	0.4952
Age	0.0493 (1, 77.7964)	0.0412	-0.3073 to 0.4123	0.8249
Sex (M=1, F=0)	0.0703 (1, 72.1810)	0.1016	-0.6163 to 0.8438	0.7916
Infections since LC onset	0.0230 (1, 69.2800)	-0.0295	-0.4037 to 0.3755	0.8798
VOC wave	1.0019 (3, 71.8813)			0.3971

**Sit-to-Stand**				
**Predictor**	**F (df1, df2)**	**Estimate**	**95% CI**	**P**
Comorbidity	0.1948 (1, 105.0000)	1.4477	-4.8471 to 7.9192	0.6598
Time since last infection (wk)	0.1598 (1, 105.0000)	-0.5703	-3.3318 to 2.2614	0.6902
Number of vaccinations	0.0165 (1, 105.0000)	0.1325	-1.7183 to 2.0435	0.8980
Age	0.0300 (1, 105.0000)	-0.1791	-2.2784 to 1.7360	0.8628
Sex (M=1, F=0)	0.0030 (1, 105.0000)	-0.1127	-4.0655 to 3.8068	0.9564
Infections since LC onset	2.3320 (1, 105.0000)	-1.5764	-3.5202 to 0.4445	0.1297
VOC wave	0.1913 (3, 105.0000)			0.9021

**KIDSCREEN-10**				
**Predictor**	**F (df1, df2)**	**Estimate**	**95% CI**	**P**
Comorbidity	0.7976 (1, 66.6497)	-3.0969	-9.9059 to 3.3736	0.3750
Time since last infection (wk)	5.6512 (1, 127.5320)	2.4870	0.4585 to 4.7356	0.0189
Number of vaccinations	0.3752 (1, 70.3257)	-0.6381	-2.6421 to 1.2969	0.5421
Age	0.1711 (1, 75.2767)	-0.4150	-2.3034 to 1.5889	0.6803
Sex (M=1, F=0)	0.0547 (1, 71.3058)	-0.4812	-4.5413 to 3.5913	0.8157
Infections since LC onset	2.2721 (1, 68.7965)	1.5673	-0.3321 to 3.4805	0.1363

VOC wave	1.3854 (3, 72.1633)			0.2541

**Parent-reported symptoms**				
**Predictor**	**F (df1, df2)**	**Estimate**	**95% CI**	**P**
Comorbidity	0.3839 (1, 61.7676)	3.1457	-6.8637 to 13.3701	0.5378
Time since last infection (wk)	3.2688 (1, 65.0432)	-2.4876	-5.3153 to 0.2793	0.0752
Number of vaccinations	0.0733 (1, 63.5671)	-0.4193	-3.3004 to 2.7563	0.7875
Age	0.7672 (1, 74.0777)	1.2872	-1.5525 to 4.1955	0.3839
Sex (M=1, F=0)	2.2378 (1, 66.4193)	-4.4794	-9.9808 to 1.0259	0.1394
Infections since LC onset	0.7852 (1, 63.8024)	-1.3765	-4.4935 to 1.4554	0.3789
VOC wave	1.1118 (3, 68.3231)			0.3504

**Patient-reported symptoms**				
**Predictor**	**F (df1, df2)**	**Estimate**	**95% CI**	**P**
Comorbidity	0.9669 (1, 59.1974)	5.4884	-6.1590 to 16.4502	0.3295
Time since last infection (wk)	0.6565 (1, 65.5877)	-1.3456	-4.5342 to 1.9493	0.4207
Number of vaccinations	0.1125 (1, 59.7655)	-0.5829	-4.0278 to 2.9011	0.7385
Age	6.4961 (1, 73.2604)	4.7332	0.8450 to 8.2929	0.0129
Sex (M=1, F=0)	1.7257 (1, 61.6299)	-4.4647	-11.2433 to 2.3144	0.1938
Infections since LC onset	0.0624 (1, 62.4659)	-0.4405	-3.8429 to 2.8847	0.8036
VOC wave	0.1761 (3, 64.4650)			0.9122

**CSHQ**				
**Predictor**	**F (df1, df2)**	**Estimate**	**95% CI**	**P**
Comorbidity	0.1534 (1, 67.0516)	1.0812	-4.0973 to 6.5358	0.6965
Time since last infection (wk)	2.3962 (1, 113.9604)	-1.2340	-2.8364 to 0.3266	0.1244
Number of vaccinations	0.4726 (1, 68.6310)	-0.5639	-2.2104 to 1.0328	0.4941
Age	9.6150 (1, 74.7450)	-2.4777	-3.9515 to -0.9407	0.0027
Sex (M=1, F=0)	0.0233 (1, 70.6122)	-0.2473	-3.2816 to 2.8808	0.8792
Infections since LC onset	0.3570 (1, 67.3047)	0.4887	-1.1410 to 2.0781	0.5522
VOC wave	1.4520 (3, 69.9846)			0.2351

Supplementary Table 1c | No differences in systemic cytokine concentrations between control subgroups. Controls with CF (n = 14) were compared with controls without CF (n = 13). Two-sided Mann–Whitney U test P values (P), U statistics (U), rank-biserial correlations (r_rb), and Holm–Bonferroni-adjusted P values are given for all cytokines included in this comparison. The significance concentration was set at α = 0.05. Adjusted P values (Adj. P) were capped at 1.0.

**Cytokine**	U statistics	P	r_rb	Adj. P
**IL-1β**	85.0	0.7882	-0.0659	1.0000
**GM-CSF**	90.0	0.9797	-0.0110	1.0000
**IL-11**	80.0	0.6030	-0.1209	1.0000
**IL-12p40**	81.0	0.6445	-0.1099	1.0000
**IL-12p70**	83.0	0.7158	0.0879	1.0000
**IL-15**	75.5	0.4666	-0.1703	1.0000

Cytokine	U statistics	P	r_rb	Adj. P
**IL-18**	75.0	0.4583	0.1758	1.0000
**IL-23**	70.0	0.3195	0.2308	1.0000
**IL-27**	51.0	0.0543	-0.4396	1.0000
**IL-5**	77.5	0.5218	0.1484	1.0000
**IL-13**	76.5	0.478	-0.1593	1.0000
**IL-6**	82.0	0.6618	0.0989	1.0000
**IL-10**	66.0	0.2293	0.2747	1.0000
**IFNγ**	88.5	0.3400	-0.1868	1.0000
**TNFα**	71.0	0.9226	0.0275	1.0000
**IL-22**	62.0	0.3438	0.2198	1.0000
**IFNα**	62.0	0.1665	-0.3187	1.0000
**IL-1α**	78.0	0.5252	0.1429	1.0000

Supplementary Table 1d | Full analysis and sensitivity analysis of lead cytokines using stratified control subgroups. Related to Table 2 of the main text. Predefined lead cytokines, one representing each functional cytokine family, are shown for pairwise comparisons between paediatric LC subgroups stratified by time since index infection and different control subgroups. LC subgroups were defined as LC < 1 year and LC 1–3 yr. The top panels show comparisons of all controls combined (healthy + CF) versus LC subgroups and healthy controls versus LC subgroups, whereas the lower panel shows comparisons of clinically stable cystic fibrosis (CF) controls versus LC subgroups. Arrows indicate the direction of change relative to the respective control subgroup (↑ higher in LC, ↓ lower in LC, ↔ no difference). These analyses were performed to assess the robustness of the pooled-control comparisons reported in Table 2. Overall group differences were assessed using two-sided Kruskal–Wallis tests, followed by unadjusted Dunn's post hoc pairwise comparisons, where applicable, versus the respective control subgroup. P values are reported for each comparison. Holm–Bonferroni adjustment for multiple testing was applied across all five lead cytokines within each pairwise comparison set, and adjusted P values are shown. H statistics, 95% confidence intervals for each subgroup, Dunn's Z values, and effect sizes (E) are reported.

All controls (healthy + CF) versus LC subgroups.

Functional families	Lead cytokines	H statistic	95% CI control	95% CI LC < 1 yr	95% CI LC 1–3 yr
**SARS-CoV-2-related**	IL-13	18.33	14.88 to 32.27	17.96 to 47.90	11.95 to 30.08
**Th2/Th1-like balance**	IL-4/IFNγ ratio	28.28	0.095 to 0.201	0.318 to 0.448	0.271 to 0.372
**Innate-related**	IL-1β	23.62	2.334 to 13.08	4.965 to 14.13	6.190 to 16.41
**Regulatory-related**	IL-11	12.32	7.102 to 12.27	13.42 to 26.46	11.22 to 23.83
**Th17/22-related**	IL-12p40	23.60	372.9 to 1039	668.8 to 1552	971.4 to 1770

**Functional families**	**Lead cytokines**	**P, direction LC < 1 yr**	**Dunn's Z LC < 1 yr**	**E LC < 1 yr**	**Adj. P LC < 1 yr**	**P, direction LC 1–3 yr**	**Dunn's Z LC 1–3 yr**	**E, LC 1–3 yr**	**Adj. P LC 1–3 yr**
**SARS-CoV-2-related**	IL-13	0.0242 ↑	2.508	0.2894	**0.048**	0.2466 ↔	1.159	0.4926	0.2466
**Th2/Th1-like balance**	IL-4/IFNγ ratio	<0.0001 ↑	5.222	0.6070	**0.0005**	<0.0001 ↑	4.070	0.4796	**0.0005**
**Innate-related**	IL-1β	0.0294 ↑	2.178	0.2515	**0.0294**	0.002 ↑	4.757	0.5570	**0.034**
**Regulatory-related**	IL-11	0.001 ↑	3.500	0.4041	**0.004**	0.017 ↑	2.386	0.2793	**0.034**
**Th17/22-related**	IL-12p40	0.0102 ↑	2.570	0.2969	**0.0306**	0.0002 ↑	4.827	0.5650	**0.0008**

Healthy controls versus LC subgroups

**Functional families**	Lead cytokines	H statistic	95% CI Healthy controls	95% CI LC < 1 yr	95% CI LC 1–3 yr
**SARS-CoV-2-related**	IL-13	17.22	14.03 to 34.06	17.96 to 47.90	11.95 to 30.08
**Th2/Th1-like balance**	IL-4/IFNγ	13.65	0.075 to 0.275	0.318 to 0.448	0.271 to 0.372
**Innate-related**	IL-1β	15.40	0.537 to 21.17	4.965 to 14.13	6.190 to 16.41
**Regulatory-related**	IL-11	2.986	7.360 to 15.96	13.42 to 26.46	11.22 to 23.83
**Th17/22-related**	IL-12p40	10.92	323.1 to 1593	668.8 to 1552	971.4 to 1770

**Functional families**	Lead cytokines	P & direction LC <1 yr	Dunn's Z LC < 1 yr	E LC < 1 yr	Adj. P LC <1 yr	P & direction LC 1–3 yr	Dunn's Z LC 1–3 yr	E LC 1–3 yr	Adj. P LC 1–3 yr
**SARS-CoV-2-related**	IL-13	0.2143 ↑	1.242	0.1590	0.5826	0.1047 ↓	1.623	0.2113	0.2094
**Th2/Th1-like balance**	IL-4/IFNγ	0.00021 ↑	3.690	0.4725	**0.0011**	0.0053 ↑	2.788	0.3629	**0.0196**
**Innate-related**	IL-1β	0.1942 ↑	1.298	0.1662	0.5826	0.0008 ↑	3.351	0.4362	**0.0040**
**Regulatory-related**	IL-11	0.0694 ↑	1.816	0.2325	0.2776	0.3356 ↑	0.9629	0.1254	0.3356
**Th17/22-related**	IL-12p40	0.3118 ↑	1.011	0.1295	0.5826	0.0049 ↑	2.813	0.3662	**0.0196**

CF controls versus LC subgroups.

**Functional families**	Lead cytokines	H statistic	95% CI CF controls	95% CI LC < 1 yr	95% CI LC 1–3 yr
**SARS-CoV-2-related**	IL-13	19.33	6.805 to 40.47	17.96 to 47.90	11.95 to 30.08
**Th2/Th1-like balance**	IL-4/IFNγ	21.43	0.081 to 0.156	0.318 to 0.448	0.271 to 0.372
**Innate-related**	IL-1β	19.50	1.887 to 8.787	4.965 to 14.13	6.190 to 16.41
**Regulatory-related**	IL-11	12.90	4.620 to 11.26	13.42 to 26.46	11.22 to 23.83
**Th17/22-related**	IL-12p40	24.55	216.6 to 745.0	668.8 to 1552	971.4 to 1770

Functional families	Lead cytokines	P & direction LC <1 yr	Dunn's Z LC < 1 yr	E LC < 1 yr	Adj. P LC < 1 yr	P & direction LC 1–3 yr	Dunn's Z LC 1–3 yr	E LC 1–3 yr	Adj. P LC 1–3 yr
**SARS-CoV-2-related**	IL-13	0.0154 ↑	2.424	0.3129	**0.0308**	0.7146 ↔	0.3657	0.0480	0.7146
**Th2/Th1-like balance**	IL-4/IFNγ	<0.0001 ↑	4.496	0.5805	**0.0005**	0.0003 ↑	3.617	0.4750	**0.0009**
**Innate-related**	IL-1β	0.0644 ↑	1.850	0.2388	0.0644	0.0001 ↑	3.843	0.5047	**0.0005**
**Regulatory-related**	IL-11	0.0006 ↑	3.451	0.4456	**0.0024**	0.0089 ↑	2.615	0.3434	**0.0178**
**Th17/22-related**	IL-12p40	0.0053 ↑	2.789	0.3597	**0.0159**	<0.0001 ↑	4.529	0.5945	**0.0005**

Supplementary Table 2.1 | LMM analysis of FEV1 z-scores. Related to Fig. 2d.

Predisposing factors for paediatric LC do not associate with lung function, as determined by FEV1 z-score, over the disease course. Two-sided linear mixed-effects model (LMM) was fitted with FEV1 z-score as the outcome. Fixed effects included comorbidity, number of vaccinations prior to LC onset, age, sex, SARS-CoV-2 variant-of-concern (VOC) wave, TsinceIndex (cut-off 52 weeks), and number of infections since LC onset; participant ID was modelled as a random intercept. Degrees of freedom were approximated using Satterthwaite's method, and 95% confidence intervals were estimated using the Wald method. Continuous covariates were standardized; categorical predictors were modelled as factors. Model fit statistics (n = 131 observations) are: conditional R^2 = 0.7318 and marginal R^2 = 0.0757.

Predictors	F (df1, df2)	Estimate	95% CI	raw P
Comorbidity	0.6858 (1, 65.4774)	-0.3103	-1.0521 to 0.4315	0.4106
Vaccination number prior to LC onset	0.0137 (1, 69.7572)	-0.0131	-0.2339 to 0.2077	0.9071
Age	0.0401 (1, 62.8733)	0.0222	-0.1972 to 0.2416	0.8420
Sex (M=1, F=0)	0.5985 (1, 63.9730)	0.1721	-0.2683 to 0.6126	0.4420
VOC wave	1.0831 (3, 62.2966)	omnibus test	see contrasts below	0.3629
TsinceIndex	1.5049 (1, 100.9152)	-0.1777	-0.4646 to 0.1091	0.2228
Infections since LC onset	3.0659 (1, 64.4461)	0.1995	-0.0261 to 0.4251	0.0847

VOC wave contrast	Estimate	95% CI	raw P
2 vs 3	0.0799	-0.4061 to 0.5660	0.7458
1 vs 3	0.8880	-0.1643 to 1.9404	0.0998
0 vs 3	-0.2387	-1.2853 to 0.8079	0.6531

Supplementary Table 2.2 | a Base linear mixed-effects model for FEV1 (force expiratory volume in 1 sec) z-score. Related to Fig. 2d. A two-sided linear mixed-effects model (LMM) was used to assess associations of IL-6 and IL-13 with FEV1 z-score in paediatric LC, with participant ID included as a random intercept to account for repeated measurements. Model fit (n = 131 observations) showed marginal and conditional R^2 values of 0.1622 and 0.7210, respectively, and was significant by likelihood-ratio testing ($P < 0.0001$). Degrees of freedom were approximated using Satterthwaite's method, and 95% confidence intervals were obtained by percentile bootstrap (1,000 resamples). No multiple-comparison adjustment was applied.

Predictor	F (df1, df2)	Estimate	95% CI	raw P
IL-6	9.0981 (1, 125.1064)	-0.4432	-0.7339 to -0.1524	0.0031
IL-13	19.2841 (1, 126.6395)	0.6806	0.3739 to 0.9873	<0.0001

b Confounder-adjusted LMM for FEV1 z-score. Related to Fig. 2d. A two-sided linear mixed-effects model (LMM) was used to assess associations of IL-6 and IL-13 with FEV1 z-score in paediatric LC after adjustment for comorbidity, SARS-CoV-2 VOC wave, number of infections prior to LC onset, number of vaccinations, TsinceIndex, sex and age, with participant ID included as a random intercept. Model fit (n = 131 observations) showed marginal and conditional R^2 values of 0.1974 and 0.7426, respectively, and was significant by likelihood-ratio testing (P = 0.0128). Degrees of freedom were approximated using Satterthwaite's method, and 95% confidence intervals were obtained by percentile bootstrap (1,000 resamples). No multiple-comparison adjustment was applied.

Predictor	F (df1, df2)	Estimate	95% CI	raw P
Comorbidity	0.0915 (1, 65.2597)	-0.1065	-0.8299 to 0.5732	0.7632
Vaccination number prior to LC onset	0.1274 (1, 68.5419)	0.0380	-0.1635 to 0.2419	0.7223
Age	0.0098 (1, 60.7239)	-0.0103	-0.2090 to 0.2058	0.9216
Sex (M=1, F=0)	0.0582 (1, 61.7337)	0.0514	-0.3695 to 0.4371	0.8102
VOC wave	1.1576 (3, 60.1613)	omnibus test	see contrasts below	0.3334
TsinceIndex	0.0135 (1, 105.3448)	-0.0170	-0.3009 to 0.2741	0.9078
Infections since LC onset	1.8162 (1, 62.3162)	0.1448	-0.0730 to 0.3462	0.1826
IL-6	7.4258 (1, 113.9670)	-0.4209	-0.7345 to -0.1299	0.0074
IL-13	15.2538 (1, 117.4568)	0.6598	0.3431 to 0.9912	0.0002

VOC wave contrast	Estimate	95% CI	raw P
2 vs 3	-0.0833	-0.5454 to 0.3677	0.7248
1 vs 3	0.7972	-0.1055 to 1.7461	0.1109
0 vs 3	-0.2944	-1.2174 to 0.6858	0.5506

Supplementary Table 2.3: Systemic cytokines are increased within predefined functional cytokine families in paediatric LC. Control vs. LC (< 1 yr) and control vs LC (1–3 yr). Additional information for Fig. 2c, e–h of the main text. Cytokines were grouped into predefined functional cytokine families and compared across three cohorts: healthy controls, paediatric LC within the first year after TsinceIndex (LC < 1 yr), and paediatric LC 1–3 yr after the initial infection. For each cytokine, overall group differences were assessed using the two-sided Kruskal–Wallis test, followed by Dunn's post-hoc tests for pairwise comparisons between controls and LC < 1 yr, and between controls and LC 1–3 yr. In addition to between-family testing using lead cytokines (Table 1), within-family multiplicity was controlled using the Holm–Bonferroni method, yielding adjusted P values for each contrast (adj. P (C vs LC < 1 yr) and adj. P (C vs LC 1–3 yr)). Lead cytokines used to probe predefined biological subgroups are listed first within each functional cytokine family. Exception: Th1/Th2 ratio as IL-4/IFNγ was used as lead in B) that was significantly increased at both TsinceIndex windows compared to Control. Significant entries (adjusted P < 0.05) are shown in **bold**; * indicates increases relative to controls. Adjusted P values > 1.0000 were truncated to 1.0; E, effect size.

Cytokine	P (C vs. LC < 1 yr)	P (C vs. LC 1–3 yr)	Adj. P (C vs. LC < 1yr)	Adj. P (C vs. LC 1–3 yr)
**A) SARS-CoV-2-related cytokines**				
**IL-13**	0.0242	0.2466	**0.0484***	0.4932
**IL-6**	0.7979	0.3828	0.7979	0.3828
**IL-33**	0.0032	0.185	**0.0096***	0.5500
**B) Th2/Th1-related balance**				
**IL-4**	0.0026	0.1865	**0.0156***	0.3730
**IL-5**	0.0133	0.0002	0.0665	**0.0014**

Cytokine	P (C vs. LC < 1 yr)	P (C vs. LC 1–3 yr)	Adj. P (C vs. LC < 1yr)	Adj. P (C vs. LC 1–3 yr)
**IL-9**	0.2581	0.0361	0.616	0.1083
**IL-2**	0.0756	0.4988	0.3024	0.4988
**IFNγ**	0.0004	0.0006	**0.0028**	**0.0036**
**TNFα**	0.308	0.0152	0.616	0.0608
**IL-12p70**	0.969	0.01	0.969	0.0500
**IL-15**	0.8877	0.965	0.8319	0.9650
**IL-18**	0.4721	0.8654	0.1813	0.4957
**C) Innate-like cytokines**				
**IL-1β**	0.0294	0.002	0.0588	**0.0040***
**IL-1α**	0.0061	**0.001**	**0.0183***	**0.0030***
**GM-CSF**	0.5663	0.4488	0.5663	0.4488
**D) Regulatory cytokines**				
**IL-11**	0.001	0.017	**0.003***	**0.0340***
**IL-10**	0.0008	0.0002	**0.0024**	**0.0006**
**IL-27**	0.4262	0.6388	0.4262	0.6388
**E) Th17-associated cytokines**				
**IL-12p40**	0.0102	**0.0002**	**0.041**	**0.0010***
**IL-22**	0.0396	0.002	0.1092	**0.0060**
**IL-23**	0.1244	0.0002	0.2488	**0.0010***
**IL-17A**	0.3408	0.2976	0.3408	0.5952
**IL-17F**	0.01	0.8823	0.05	0.8823

Cytokine	H statistic	95% CI control	95% CI LC < 1 yr	95% CI LC 1–3 yr	Dunn's Z C vs. LC < 1yr	E C vs. LC < 1yr	Dunn's Z C vs. LC 1–3 yr	E C vs. LC 1–3 yr
A) SARS-CoV-2-related cytokines								
IL-13	18.33	14.88 to 32.27	17.96 to 47.90	11.95 to 30.08	2.508	0.2894	1.159	0.4926
IL-6	3.566	10.73 to 22.32	11.67 to 33.31	6.652 to 28.19	0.256	0.0296	1.307	0.1529
IL-33	10.68	14.31 to 96.09	34.28 to 106.6	22.79 to 100.7	3.164	0.3653	1.325	0.1551
B) *Th2/Th1-related balance*								
IL-4	11.08	1.200 to 35.77	5.380 to 32.11	2.541 to 35.10	3.214	0.3710	1.321	0.1546
IL-5	16.72	1.349 to 5.717	0.259 to 8.653	0.480 to 12.63	2.476	0.2859	4.088	0.4785
IL-9	14.04	3.375 to 18.19	2.300 to 18.09	2.830 to 17.41	1.131	0.1306	2.096	0.2453
IL-2	10.62	4.063 to 9.965	3.084 to 6.854	3.530 to 8.341	2.077	0.2398	0.676	0.0791
IFNγ	15.53	14.12 to 269.6	19.12 to 210.6	7.773 to 243.1	3.519	0.4063	3.578	0.4187
TNFα	7.802	35.48 to 200.6	11.84 to 406.9	73.54 to 507.3	1.020	0.1178	2.671	0.3125
IL-12p70	0.248	1.864 to 3.4	1.550 to 6.980	1.706 to 5.810	0.039	0.0045	0.365	0.0427
IL-15	0.04819	76.48 to 163.5	86.02 to 155.2	82.52 to 147.5	0.141	0.0163	0.044	0.0051
IL-18	1.807	171.1 to 724.5	104.5 to 130.8	97.70 to 123.7	0.719	0.0830	1.337	0.1565
C) Innate-like cytokines								
IL-1β	23.62	2.334 to 13.08	4.965 to 14.13	6.190 to 16.41	2.178	0.2515	4.757	0.5570
IL-1α	12.82	5.491 to 14.30	9.803 to 16.02	10.07 to 18.10	2.743	0.3167	3.508	0.4104
GM-CSF	4.353	0.689 to 1.734	0.281 to 5.231	0.714 to 3.430	0.574	0.0662	1.215	0.1422
D) Regulatory cytokines								
IL-11	12.32	7.102 to 12.27	13.42 to 26.46	11.22 to 23.83	3.500	0.4041	2.386	0.2793
IL-10	31.37	2.057 to 3.527	1.520 to 3.336	1.005 to 2.653	3.357	0.3875	5.598	0.6548
IL-27	1.042	22.16 to 61.21	22.44 to 150.7	15.28 to 154.8	0.796	0.0919	0.996	0.1166
E) Th17-associated cytokines								
IL-12p40	23.60	372.9 to 1039	668.8 to 1552	971.4 to 1770	2.570	0.2969	4.827	0.5650
IL-22	10.85	3.690 to 10.03	2.573 to 8.689	2.100 to 4.996	2.058	0.2376	3.294	0.3855
IL-23	17.74	5.104 to 19.11	8.923 to 18.35	9.395 to 21.46	1.536	0.1774	4.027	0.4712
IL-17A	1.690	0.509 to 0.836	0.523 to 0.684	0.312 to 1.241	0.953	0.1100	1.285	0.1504
IL-17F	0.102	1.076 to 7.284	0.330 to 11.07	1.384 to 6.477	0.313	0.0362	0.148	0.0173

Supplementary Table 2.4 | Comparison of systemic cytokines and autoantibodies over the course of paediatric LC. Related to Fig. 2, Fig. 3 and Supplementary Fig. 2. Serum cytokines and autoantibodies were analysed in paediatric LC patients with two visits per person. The upper table reports P values and numbers of observations from two-sided linear mixed-effects models (LMMs), with TsinceIndex entered as a binary fixed factor (LC < 1 yr versus LC 1–3 yr) and participant ID included as a random intercept. Multiple testing within functional families was controlled using the Holm–Bonferroni method; adjusted P values (Adj. P) are shown. Adj. P values > 1.0000 are reported as 1.0000. The lower table reports the corresponding model output, including F statistics, estimates, standard errors and 95% confidence intervals.

Analyte	P	Adj. P	obs (n)
**a) SARS-CoV-2-related cytokines (see Fig. 2)**			
**IL-6**	0.0353	0.0706	139
**IL-13**	< 0.0001	**0.0003**	139
**IL-33**	0.5316	1.0000	139
**b) Th1/2-related cytokines**			
**Ratio IL-4/IFNγ**	0.6099	0.6099	139
Th2-related cytokines			
**IL-4**	0.6781	1.0000	139
**IL-5**	0.6051	1.0000	139
**IL-9**	0.514	1.0000	139
Th1-related cytokines			
**IL-2**	0.3187	1.0000	139
**IFNγ**	0.3683	1.0000	139
**TNFα**	0.4887	1.0000	139
**IL-12p70**	0.4736	1.0000	139
**IL-15**	0.1183	0.7098	139
**IL-18**	0.5074	1.0000	139
**c) Regulatory cytokines**			
**IL-11**	0.0161	**0.0322**	139
**IL-10**	0.0065	**0.0195**	139
**IL-27**	0.1458	0.1458	139
**d) Innate-like cytokines**			
**IL-1β**	0.2541	0.7623	139
**IL-1α**	0.6328	1.0000	139
**GM-CSF**	0.6087	1.0000	139
**e) Th17/22-related cytokines**			
**IL-12p40**	0.1290	0.5160	139
**IL-17A**	0.7748	1.0000	139
**IL-17F**	0.2297	0.6891	139
**IL-22**	0.0044	**0.0220**	139
**IL-23**	0.6879	1.0000	139
**f) aAb *(see Fig. 2, 3)***			
**Anti-Cardiolipin**	0.0098	0.0686	139
**Anti-β-2 GPI**	0.0306	0.1020	137
**Anti-Prothrombin**	0.0138	0.0732	139
**Anti-MPO***	0.0122	0.0732	133
**Anti-PR3**	0.0255	0.1020	138
**Anti-CCP**	0.8388	0.8388	139
**Anti-TransG**	0.1318	0.2636	139
**aAb per ID (Fig.3c)**	0.5770	unadjusted	139

Analyte	F (df1, df2)	Estimate	SE	95% CI
**IL-6**	4.5645 (1, 91.5371)	9.1822	4.2978	-17.1747 to -0.9613
**IL-13**	29.2424 (1, 73.5456)	-16.3023	3.0147	-22.4205 to -9.9889
**IL-33**	0.3935 (1, 128.1152)	-14.2744	22.7546	-61.2701 to 30.2500
**IL-4/IFNγ**	0.2615 (1, 135.2056)	-0.0187	0.0365	-0.0885 to 0.0474
**IL-4**	0.1735 (1, 84.6014)	-2.1709	5.2117	-12.2446 to 7.7826
**IL-5**	0.1819 (1, 129.2504)	1.9539	4.5812	-7.1686 to 10.5094
**IL-9**	0.2695 (1, 80.0390)	-1.2108	2.3322	-5.9688 to 3.0850
**IL-2**	1.0067 (1, 81.7885)	0.7194	0.7170	-0.7447 to 2.1107
**IFNγ**	0.8196 (1, 71.9630)	-21.9319	24.2263	-65.4031 to 29.4766
**TNFα**	0.4820 (1, 131.7864)	90.9380	130.9835	-163.8169 to 367.4913
**IL-12p70**	0.5180 (1, 89.6848)	-0.6800	0.9447	-2.6584 to 1.1659
**IL-15**	2.4784 (1, 109.1315)	-26.4264	16.7862	-61.4815 to 9.1382
**IL-18**	0.4417 (1, 135.3279)	-5.8488	8.8000	-22.8599 to 11.6678
**IL-11**	6.1318 (1, 89.5571)	-5.8335	2.3558	-10.2175 to -1.3949
**IL-10**	7.7386 (1, 98.5442)	-1.0698	0.3846	-1.8105 to -0.2991
**IL-27**	2.1601 (1, 75.6540)	-25.4205	17.2961	-59.1071 to 11.6122
**IL-1α**	0.2296 (1, 100.7445)	-0.7987	1.6667	-3.9619 to 2.3416
**IL-1β**	1.3204 (1, 77.5945)	1.5991	1.3917	-0.8991 to 4.4210
**GM-CSF**	0.2641 (1, 84.2039)	-0.3600	0.7005	-1.7576 to 0.9291
**IL-12p40**	2.3463 (1, 91.8922)	263.0140	171.7084	-71.3675 to 583.5334
**IL-17A**	0.0822 (1, 126.8193)	0.0593	0.2068	-0.3612 to 0.4666
**IL-17F**	1.4638 (1, 84.4674)	-1.7680	1.4614	-4.6962 to 1.0345
**IL-22**	8.5028 (1, 94.6352)	-2.9107	0.9982	-4.9782 to -1.0066
**IL-23**	0.1624 (1, 92.2683)	0.8976	2	-3.4798 to 5.3329
**Anti-Cardiolipin**	7.0677 (1, 88.9909)	-0.9769	0.3675	-1.6947 to -0.2955
**Anti-β-2 GPI**	4.9779 (1, 128.1020)	1.8438	0.8264	0.2884 to 3.4068

Analyte	F (df1, df2)	Estimate	SE	95% CI
**Anti-Prothrombin**	6.1623 (1, 91.3899)	-1.5283	0.6156	-2.7908 to -0.3558
**Anti-MPO**	6.3357 (1, 125.8138)	-0.2230	0.0886	-0.4014 to -0.0646
**Anti-PR3**	5.0049 (1, 136.0000)	0.5074	0.2268	0.0625 to 0.9525
**Anti-CCP**	0.0377 (1, 80.3247)	0.0959	0.4938	-0.8992 to 1.0445
**Anti-TransG**	1.7493 (1, 95.0376)	1.4222	1.0753	-0.6459 to 3.5138
**aAB reactivity per ID Fig. 3c**	0.3212(1;100.08)	0.0397	0.0701	−0.0960 to 0.1700

Supplementary Table 2.5a | Primary model predicting Bell score severity. Related to Fig. 2k. A two-sided linear mixed-effects model (LMM) was used to relate Bell score to IL-12p40, mean corpuscular haemoglobin concentration (MCHC), absolute basophil granulocyte counts and anti-EBV EBNA titres in paediatric LC, with participant ID included as a random intercept to account for repeated measurements. All continuous variables were z-standardized. The model showed marginal and conditional R^2 values of 0.2121 and 0.7858, respectively, and was significant by likelihood-ratio testing ($P < 0.0001$); fixed effects were evaluated using Satterthwaite's method. No multiple-comparison adjustment was applied.

Predictor	F (df1, df2)	Estimate	95% CI	raw P
IL-12p40	16.8695 (1, 103.0928)	7.6798	3.8605 to 11.1478	<0.0001
MCHC	7.4328 (1, 109.7204)	-4.9544	-8.4997 to -1.5698	0.0075
Basophil granulocytes (absolute)	3.9514 (1, 109.5586)	3.5241	0.0279 to 6.7380	0.0493
EBV EBNA IgG	5.8558 (1, 67.8263)	-5.5295	-9.7982 to -0.8609	0.0182

Supplementary Table 2.5b | Extended confounder-adjusted interaction model predicting Bell score severity. Related to Fig. 2k. A two-sided linear mixed-effects model (LMM) was used to relate Bell score to IL-12p40, MCHC, absolute basophil granulocyte counts and anti-EBV EBNA titres in paediatric LC, adjusted for age, sex, comorbidity status and TsinceIndex, with additional interaction terms between anti-EBV EBNA and MCHC, vitamin B1, IL-12p40 and basophil counts. Participant ID was included as a random intercept. Model fit is reported as marginal and conditional R^2 (R^2m = 0.3328, R^2c = 0.8303), with model significance confirmed by likelihood-ratio testing ($P < 0.0001$); fixed effects were evaluated using Satterthwaite's method. No multiple-comparison adjustment was applied.

Predictor	F (df1, df2)	Estimate	95% CI	raw P
Comorbidity	0.4984 (1, 52.2331)	-5.3258	-20.3005 to 9.6488	0.4833
Sex (M=1, F=0)	0.3196 (1, 61.1489)	2.6716	-6.7091 to 12.0523	0.5739
Age	2.8688 (1, 54.3053)	3.7375	-0.6426 to 8.1177	0.0960
IL-12p40	13.7416 (1, 93.0773)	8.0414	3.7354 to 12.3474	0.0004
MCHC	10.4664 (1, 97.8929)	-6.0036	-9.6872 to -2.3200	0.0017
Basophil granulocytes	4.1279 (1, 97.1284)	3.5704	0.0822 to 7.0587	0.0449
Anti-EBV EBNA	2.5279 (1, 58.6863)	-3.7304	-8.3878 to 0.9269	0.1172
Vitamin B1	2.3614 (1, 78.3436)	2.4279	-0.7083 to 5.5641	0.1284
TsinceIndex	0.3677 (1, 96.9658)	-1.1746	-5.0196 to 2.6704	0.5457
Basophil granulocytes × Anti-EBV EBNA	0.5291 (1, 97.4735)	-1.3575	-5.0621 to 2.3470	0.4687
Anti-EBV EBNA × Vitamin B1	6.5743 (1, 66.3103)	-3.6861	-6.5398 to -0.8325	0.0126
MCHC × Anti-EBV EBNA	6.2641 (1, 97.9603)	4.4452	0.9197 to 7.9706	0.0140
IL-12p40 × Anti-EBV EBNA	2.7041 (1, 97.9408)	-2.9667	-6.5478 to 0.6144	0.1033

Supplementary Table 3.1 | No relevant correlations between G-protein-specific autoantibodies (aAb) and disease severity or cytokines. Additional information for the main text. Two-sided linear mixed-effects models (LMM) were used to account for repeated patient visits. G-protein-specific autoantibodies (aAb, U mL^{-1}) were used as dependent variables; predictors are listed in rows. Participant ID was included as a random intercept. Cytokines were selected based on significant differences between the Control and LC groups (Fig. 2c,f–h). Marginal R^2 (R^2m) and the corresponding P value for the fixed effect are reported. No association was both statistically significant and associated with $R^2m > 0.20$. Including age and time since index infection as covariates, and sex and vaccination status (Y/N) as factors, did not yield any model that was both statistically significant and associated with $R^2m > 0.20$.

**Predictor**	β1-adrenergic receptor aAb	β2-adrenergic receptor aAb	M3 muscarinic receptor	M4 muscarinic receptor	
Bell score	0.0062<br>0.3056	0.0130<br>0.1779	0.0001<br>0.8994	0.0081<br>0.2477	R²m<br>P
TsinceIndex	0.0023<br>0.5215	0.0029<br>0.5284	0.0001<br>0.9548	0.0192<br>0.0684	R²m<br>P
IL-6	0.0002<br>0.8752	0.0011<br>0.7117	0.0041<br>0.5086	0.0082<br>0.3013	R²m<br>P
IL-13	0.0028<br>0.5900	0.0007<br>0.7983	0.0060<br>0.4493	0.0292<br>0.0843	R²m<br>P
IFNγ	0.0000<br>0.9620	0.0008<br>0.7949	0.0018<br>0.6919	0.0034<br>0.5810	R²m<br>P
IL-1α	0.0073<br>0.2556	0.0122<br>0.1837	0.0134<br>0.2004	0.0232<br>0.0551	R²m<br>P
IL-1β	0.0161<br>0.1845	0.0520<br>0.0717	0.0268<br>0.1056	0.0080<br>0.3613	R²m<br>P
GM-CSF	0.0026<br>0.5805	0.0100<br>0.2976	0.0137<br>0.2410	0.0012<br>0.7091	R²m<br>P
IL-11	0.0023<br>0.5616	0.0028<br>0.5553	0.0177<br>0.1620	0.0230<br>0.0784	R²m<br>P
IL-12p40	0.0056<br>0.3557	0.0179<br>0.1265	0.0233<br>0.0999	0.0011<br>0.6887	R²m<br>P
IL-33	0.0058<br>0.2203	0.0137<br>0.0989	0.0120<br>0.1778	0.0050<br>0.2872	R²m<br>P

anti-β1-AdrR aAb

**Predictor**	obs	F (df1, df2)	Estimate	95% CI
Bell score	119	1.0618 (1, 97.2937)	1.0898	-1.0051 to 3.1847
TsinceIndex	140	0.4061 (1, 126.7168)	-0.6625	-2.7187 to 1.3936
IL-6	137	0.0233 (1, 134.5209)	0.1782	-2.1523 to 2.5713
IL-13	137	0.2919 (1, 119.9872)	0.7299	-1.9955 to 3.5498
IFNγ	137	0.0022 (1, 102.6684)	0.0674	-2.7027 to 3.0731
IL-1α	137	1.2846 (1, 128.5779)	1.1822	-0.8783 to 3.1463
IL-1β	137	1.7522 (1, 120.2470)	1.7545	-0.7460 to 4.2830
GM-CSF	137	0.3069 (1, 132.2081)	0.6969	-1.7108 to 3.1392
IL-11	137	0.3293 (1, 134.6503)	0.6598	-1.5594 to 2.9780
IL-12p40	137	0.8408 (1, 134.6275)	1.0262	-1.0468 to 3.2474
IL-33	137	1.4879 (1, 103.5502)	1.0437	-0.7055 to 2.6174

anti-β2-AdrR aAb

**Predictor**	obs	F (df1, df2)	Estimate	95% CI
Bell score	120	1.8374 (1, 111.6144)	1.7061	-0.7868 to 4.1991
TsinceIndex	140	0.4279 (1, 136.5683)	0.8061	-1.6308 to 3.2430
IL-6	138	0.1339 (1, 131.7679)	0.5024	-2.0579 to 2.8919
IL-13	138	0.0641 (1, 105.2207)	0.3856	-2.5850 to 3.5281
IFNγ	138	0.0680 (1, 91.5607)	0.4146	-2.5795 to 3.5294
IL-1α	138	1.7607 (1, 135.9993)	1.6544	-0.7283 to 4.0840
IL-1β	138	3.2872 (1, 107.0304)	2.7045	-0.1645 to 5.5603
GM-CSF	138	1.0761 (1, 120.3952)	1.4985	-1.3897 to 4.2823
IL-11	138	0.3406 (1, 131.6819)	0.7873	-1.9664 to 3.2713

Predictor	obs	F (df1, df2)	Estimate	95% CI
IL-12p40	138	2.3177 (1, 132.2712)	1.9943	-0.5342 to 4.3968
IL-33	138	2.7233 (1, 120.3719)	1.7508	-0.2745 to 3.9425

anti-M3-mAChR aAb

Predictor	obs	F (df1, df2)	Estimate	95% CI
Bell score	120	0.0150 (1, 117.5213)	-0.1443	-2.4757 to 2.1871
TsinceIndex	140	0.0074 (1, 132.6103)	0.0968	-2.1321 to 2.3258
IL-6	138	0.4335 (1, 113.8934)	0.7982	-1.6857 to 3.1956
IL-13	138	0.5652 (1, 89.9698)	0.9668	-1.5233 to 3.3499
IFNγ	138	0.1594 (1, 80.8118)	0.5276	-2.1654 to 3.1087
IL-1α	138	1.6369 (1, 127.3435)	1.4448	-0.7266 to 3.5207
IL-1β	138	2.6287 (1, 91.8670)	2.0516	-0.5288 to 4.5293
GM-CSF	138	1.3751 (1, 101.6193)	1.4646	-1.0900 to 3.8232
IL-11	138	1.9397 (1, 114.3462)	1.6497	-0.7011 to 4.0528
IL-12p40	138	2.6913 (1, 116.8572)	1.9003	-0.3768 to 4.1429
IL-33	138	1.8013 (1, 134.8442)	1.3600	-0.6851 to 3.5155

anti-M4-mAChR aAb

Predictor	obs	F (df1, df2)	Estimate	95% CI
Bell score	120	1.3298 (1, 101.4168)	-1.0069	-2.7364 to 0.7225
TsinceIndex	140	3.3269 (1, 129.8961)	-1.5797	-3.2924 to 0.1330
IL-6	138	1.0607 (1, 135.5846)	1.0322	-0.9883 to 3.1290
IL-13	138	3.0340 (1, 113.8515)	1.9755	-0.3771 to 4.2279
IFNγ	138	0.3036 (1, 95.9690)	0.6622	-1.7717 to 2.8883
IL-1α	138	3.7494 (1, 134.0628)	1.7368	-0.0331 to 3.4683
IL-1β	138	0.8214 (1, 111.7070)	1.0131	-1.2164 to 3.2468
GM-CSF	138	0.1357 (1, 127.2118)	0.3951	-1.7082 to 2.5099
IL-11	138	3.0850 (1, 135.4435)	1.7180	-0.2835 to 3.7670
IL-12p40	138	0.1574 (1, 135.4022)	0.3835	-1.5937 to 2.3573
IL-33	138	1.1181 (1, 112.2391)	0.8038	-0.6704 to 2.2476

Supplementary Table 3.2. GPCR autoantibodies across the course of paediatric long COVID. Serum cytokines and functional G protein-coupled receptor (GPCR) autoantibodies (aAb) were analysed in paediatric long COVID (LC) patients at two visits per participant. Two-sided linear mixed models were fitted with time since index infection (TsinceIndex) as a binary factor (LC < 1 yr versus LC 1–3 yr). Shown are the number of observations (obs), marginal R^2 (R^2m), F statistic with degrees of freedom, fixed-effect estimate, standard error (SE), 95% confidence interval (CI) and unadjusted and Holm–Bonferroni-adjusted P values. Confidence intervals were derived by percentile bootstrap (1,000 resamples). Adj. P values >1.0000 are reported as 1.0000. P < 0.05 was considered statistically significant.

Analyte	obs	R^2m	F (df1, df2)	Estimate	95% CI	P	Adj. P
**anti-M3-mAChR**	140	0.0016	0.2951 (1, 122.38)	1.0043	−2.6345 to 4.5513	0.5872	1.0000
**anti-M4-mAChR**	140	0.0008	0.2471 (1, 93.71)	-0.6454	-3.2826 to 1.8761	0.415	1.0000
**anti-β1-AdrR**	139	0.0015	0.4982 (1, 90.66)	1.0671	-1.9083 to 3.9106	0.4821	1.0000
**anti-β2-AdrR**	140	0.0005	0.1334 (1, 104.42)	0.6916	-3.1963 to 4.5657	0.7157	1.0000

Supplementary Table 3.3 | Autoantibody (aAb) concentrations in paediatric LC and sensitivity analyses using stratified controls (see Fig. 2, 3 and Supplementary Fig. 4 for visualisation). AAb were compared between controls and paediatric LC stratified by time since index infection (LC < 1 year; LC 1–3 yr) as additional information for Fig. 2e and Fig. 3b and c. a, Pooled controls (healthy + clinically stable cystic fibrosis (CF)) versus LC subgroups. b, Healthy controls versus LC subgroups (sensitivity analysis). c, CF controls versus LC subgroups (sensitivity analysis). Group medians and 95% confidence intervals are shown. Overall group differences were assessed using two-sided Kruskal–Wallis tests; H statistics are reported. Pairwise comparisons (control vs LC < 1 year; control vs LC 1–3 yr) were performed using Dunn's post hoc tests; exact P values, Dunn's Z statistics, effect sizes, and Holm–Bonferroni-adj. P values are shown; E, effect size. Significant entries are highlighted in bold; P values exceeding 1.0000 were set to 1.0000.

a) Pooled controls versus LC subgroups (sensitivity); (see Fig. 2, 3; Supplementary Fig. 4)

**aAb** *(see Fig. 2, 3)*	H statistic	95% CI control	95% CI LC < 1 yr	95% CI LC 1–3 yr
**Anti-Cardiolipin**	2.177	3.3 to 7.0	3.5 to 4.65	3.35 to 4.35
**Anti-β-2 GPI**	3.621	2.4 to 3.9	2.2 to 3.4	2.6 to 4.3
**Anti-Prothrombin**	5.701	3.0 to 6.3	4.25 to 6.8	3.5 to 5.75
**Anti-MPO***	21.24	0.8 to 1.2	0.5 to 0.8	0.3 to 0.7
**Anti-PR3**	26.0	0.2 to 1.0	0.025 to 0.0695	0.562 to 1.3
**Anti-CCP**	0.2465	2.2 to 6.9	2.8 to 4.65	3.1 to 5.1
**Anti-TransG**	2.790	2.8 to 8.0	2.65 to 4.4	3.0 to 5.3
**aAB reactivity per ID**	0.2210	0.0 to 1.0	0.0	0.0 to 1.0

**aAb** **(see Fig 2,3)**	P LC < 1 yr	Dunn's Z LC < 1 yr	E LC < 1 yr	Adj. P LC < 1 yr	P LC 1–3 yr	Dunn's Z LC 1–3 yr	E LC 1–3 yr	Adj. P LC 1–3 yr
**Anti-Cardiolipin**	0.6209	1.014	0.1179	1.0000	0.2822	1.472	0.1723	1.0000
**Anti-β-2 GPI**	0.1521	1.774	0.2062	0.6395	1.0000	0.5893	0.0690	1.0000
**Anti-Prothrombin**	0.1279	1.853	0.2154	0.6395	1.0000	0.0054	0.0006	1.0000
**Anti-MPO***	**0.0020**	3.288	0.3822	**0.0140**	**<0.0001**	4.581	0.5362	**0.0007**
**Anti-PR3**	**0.0041**	3.086	0.3587	**0.0246**	0.4664	1.192	0.1395	1.0000
**Anti-CCP**	1.0	0.2081	0.0242	1.0000	1.0000	0.2175	0.0255	1.0000
**Anti-TransG**	0.1915	1.666	0.1937	0.6395	0.6765	0.9576	0.1121	1.0000
**aAB reactivity per ID**	1.0	0.1520	0.022	1.0000	1.0000	0.4407	0.0516	unadjusted

b) Healthy controls versus LC subgroups (sensitivity); (see Fig. 2, 3; Supplementary Fig. 4)

**aAb** *(see Fig. 2, 3)*	**H statistic**	**95% CI control**	**95% CI LC < 1 yr**	**95% CI LC 1–3 yr**
**Anti-Cardiolipin**	0.9352	2.8-9.0	3.5 to 4.65	3.35 to 4.35
**Anti-β-2 GPI**	4.212	2.3-7.6	2.2 to 3.4	2.6 to 4.3
**Anti-Prothrombin**	4.708	2.7-10.2	4.25 to 6.8	3.5 to 5.75
**Anti-MPO***	14.78	0.6-1.4	0.5 to 0.8	0.3 to 0.7
**Anti-PR3**	23.91	0.027-1.0	0.025 to 0.0695	0.5620 to 1.3
**Anti-CCP**	2.104	2.2-15.6	2.8 to 4.65	3.1 to 5.1
**Anti-TransG**	2.755	2.1-26.6	2.65 to 4.4	3.0 to 5.3

**aAb** **(see Fig 2,3)**	**P C < 1 yr**	**Dunn's Z C < 1 yr**	**E LC < 1 yr**	**Adj.P LC < 1 yr**	**P LC 1–3 yr**	**Dunn's Z LC 1–3 yr**	**E LC 1–3 yr**	**Adj.P LC 1–3 yr**
**Anti-Cardiolipin**	1.0000	0.5960	0.0763	1.0000	0.6890	0.9453	0.1220	1.0000
**Anti-β-2 GPI**	0.1051	1.9390	0.2483	0.63006	0.5791	1.0590	0.1367	1.0000
**Anti-Prothrombin**	1.0000	0.3585	0.0459	1.0000	0.5483	1.0940	0.1412	1.0000
**Anti-MPO***	**0.0108**	2.783	0.3563	0.0756	**0.0003**	3.8350	0.4951	**0.0021**
**Anti-PR3**	0.1719	1.7170	0.2198	0.8595	0.2149	1.6100	0.2079	1.0000
**Anti-CCP**	0.2939	1.4500	0.1857	0.8817	0.5421	1.1010	0.1421	1.0000
**Anti-TransG**	0.2030	1.6380	0.2097	0.8595	0.5554	1.0860	0.1402	1.0000

c) CF controls versus LC subgroups (sensitivity); (see Fig. 2, 3; Supplementary Fig. 4)

**aAb** *(see Fig. 2, 3)*	H statistic	95% CI control	95% CI LC < 1 yr	95% CI LC 1–3 yr
**Anti-Cardiolipin**	1.869	3.2 to 7.2	3.5 to 4.65	3.35 to 4.35
**Anti-β-2 GPI**	2.058	2.4 to 3.9	2.2 to 3.4	2.6 to 4.3
**Anti-Prothrombin**	8.6883	2.8 to 4.6	4.25 to 6.8	3.5 to 5.75
**Anti-MPO***	11.40	0.6 to 1.6	0.5 to 0.8	0.3 to 0.7
**Anti-PR3**	26.77	0.2 to 1.5	0.025 to 0.0695	0.5620 to 1.3
**Anti-CCP**	2.275	1.3 to 6.1	2.8 to 4.65	3.1 to 5.1
**Anti-TransG**	1.208	2.8 to 8.0	2.65 to 4.4	3.0 to 5.3

**aAb** *(see Fig. 2, 3)*	H statistic		95% CI control		95% CI LC < 1 yr		95% CI LC 1–3 yr	
**aAb (see Fig 2.3)**	P C < 1 yr	Dunn's Z C < 1 yr	E C < 1 yr	Adj. P C < 1 yr	P LC 1–3 yr	Dunn's Z LC 1–3 yr	E LC 1–3 yr	Adj. P LC 1–3 yr
**Anti-Cardiolipin**	0.6307	1.004	0.1296	1.0	0.3455	1.363	0.1774	1.0
**Anti-β-2 GPI**	0.8668	0.7834	0.1011	1.0	1.0	1.376	0.1791	1.0
**Anti-Prothrombin**	**0.0191**	2.591	0.3345	0.1146	0.5003	1.15	0.1497	1.0
**Anti-MPO***	**0.0394**	2.332	0.3011	0.1970	**0.0016**	3.346	0.4356	**0.0112**
**Anti-PR3**	**0.0040**	3.09	0.3989	**0.0280**	1.0	0.1751	0.0228	1.0
**Anti-CCP**	0.4785	1.177	0.1520	1.0	0.2629	1.508	0.1963	1.0
**Anti-TransG**	0.6836	0.9506	0.1227	1.0	1.0	0.3988	0.0519	1.0

Supplementary Table 3.4 | Association of anti-DFS70 positivity with coagulation factors, complement components, EBV serostatus, and receptor autoantibodies in paediatric LC. *Additional information for Fig. 3e of the main text and Supplementary Fig. 3c–e.*

Analysis of coagulation factors, components of the complement system and EBV-status were analysed in paediatric LC patients with two visits per person. The table reports P values from two-sided LMMs (n = 139 observations), with anti-DFS70^{pos} vs. anti-DFS70^{neg} entered as a bivariate factor and participant ID as a cluster variable. Family-wise multiplicity was controlled using the Holm–Bonferroni (listed as "adjusted P value"). Adjusted P values greater than 1.0000 are reported as and those <0.05 were considered significant. Significant entries are shown in bold. Receptors (anti-M-mAChR) and β-adrenergic receptors (anti-β-AdrR). Lower panels report the full model output.

Anti-DFS70^{pos} vs. anti-DFS70^{neg} and coagulation factors, complement system, and EBV-status *(see Fig. 3c–e)*

**Analyte**	obs (n)	R^2m	F (df1, df2)	Estimate	95% CI	P	Adj. P
vWF%	139	0.1384	13.6020 (1, 72.8142)	-42.8349	-65.8046 to 19.8652	0.0003	0.0039
vWF Activity%	139	0.1421	14.2924 (1, 73.7543)	-38.0280	-57.9214 to 18.1346	0.0003	0.0039
F VIII%	139	0.1249	13.9225 (1, 76.1260)	-34.8917	-53.3853 to 16.3981	0.0003	0.0039
aPTT	139	0.0983	9.7138 (1, 70.8476)	2.9947	1.0944 to 4.8950	0.0022	0.0220
Antithrombin	133	0.0507	4.6834 (1, 67.0093)	8.2020	0.7034 to 15.7005	0.0311	0.2799
Fibrinogen	139	0.0099	0.8834 (1, 69.0565)	-0.1660	-0.5152 to 0.1833	0.3442	1.0000
D-Dimer	139	0.0070	0.9371 (1, 76.3026)	-27.2135	-82.8094 to 28.3824	0.3304	1.0000
Protein C	138	0.0233	1.9886 (1, 72.5387)	-9.5804	-23.0174 to 3.8566	0.1566	1.0000
Free Protein S	138	0.0163	1.2370 (1, 72.0504)	-7.1281	-19.1019 to 4.5006	0.2633	1.0000
Anti-EBV EBNA	138	0.0071	0.5563 (1, 71.1154)	-63.9302	-230.5483 to 107.8559	0.4543	1.0000
IgG EBV VCA	138	0.0026	0.2076 (1, 71.0883)	-37.6163	-200.9171 to 125.6846	0.6520	1.0000
C3	135	0.0121	1.0244 (1, 72.2856)	-0.0667	-0.1971 to 0.0637	0.3084	1.0000
C4	135	0.0004	0.0301 (1, 71.4311)	0.0031	-0.0325 to 0.0387	0.8858	1.0000

Related to Supplementary Fig. 3e

**Analyte**	obs (n)	R^2m	F (df1, df2)	Estimate	95% CI	P	Adj. P
anti-M3-mAChR	140	0.0000	0.0021 (1, 70.3001)	0.2004	-8.3500 to 8.7509	0.9268	1.0000
anti-M4-mAChR	140	0.0000	0.0015 (1, 70.5383)	0.1619	-8.0076 to 8.3314	0.9250	1.0000
anti-β1-AdrR	139	0.0007	0.0579 (1, 71.9224)	1.2121	-8.7488 to 11.1730	0.8243	1.0000
anti-β2-AdrR	140	0.0000	0.0000 (1, 72.1047)	-0.0054	-10.5810 to 10.5702	0.9209	1.0000

Supplementary Table 4 | EBV-experienced paediatric LC patients show elevated cytokine and aAb concentrations but no enhanced SARS-CoV-2-specific responses. This table provides detailed results for the two-sided LMMs shown in Table 2 and Fig. 4 of the main text. Associations of clinical scores, aAb concentrations, and cytokine concentrations with EBV exposure status were analysed in paediatric LC patients using LMMs with repeated measurements clustered by participant ID. The number of observations was n = 138 for clinical scores, anti-S Ab, and IgG CoV-2 Liaison (spike); n = 137 for anti-M3-mAChR, anti-M4-mAChR, and granulocyte subtypes; and n = 136 for anti-CCP, anti-TransG, anti- Prothrombin, and cytokine concentrations. EBV exposure status was modelled as a binary factor, distinguishing EBV-naïve patients (anti-EBV EBNA < 50 U mL^{-1}) from EBV-experienced patients (anti-EBV EBNA > 50 U mL^{-1}). Sex, vaccination status, and comorbidity were included as categorical factors, whereas time since index infection preceding LC onset and age were included as covariates. The dependent variable is indicated above each section. Holm–Bonferroni-adjusted P values are reported in Table 1 of the main text. Significant associations with EBV exposure are marked by bold numbers of the corresponding P values when the overall model fit was significant; observation (obs).

a. Anti-SARS-response

Analyte	obs (n)	Predictor	F (df1, df2)	Estimate	95% CI	raw P
**Anti-Spike**	138	TsinceIndex	0.8615 (1, 121.7985)	-312.1596	-977.5812 to 353.2621	0.3552
		Comorbidity	0.3674 (1, 68.9239)	-856.5714	-3652.6417 to 1939.4989	0.5464
		Vaccination	12.4864 (1, 71.1589)	-2739.8675	-4273.9623 to -1205.7727	0.0007
		Age	2.0782 (1, 76.8479)	573.3736	-213.5502 to 1360.2975	0.1535
		Sex (M=1, F=0)	0.0642 (1, 76.5592)	-197.6976	-1741.9398 to 1346.5446	0.8007
		EBV	0.0209 (1, 90.9542)	107.4303	-1362.5037 to 1577.3643	0.8853
**CH50**	131	TsinceIndex	1.9549 (1, 123.9267)	-1.2383	-2.9211 to 0.5924	0.1646
		Comorbidity	0.0581 (1, 68.5294)	-0.9781	-8.8457 to 6.8657	0.8102
		Vaccination	2.2468 (1, 70.2857)	3.3254	-0.5415 to 7.7925	0.1384
		Age	0.8657 (1, 78.3766)	1.0344	-1.0761 to 3.1948	0.355
		Sex (M=1, F=0)	0.9845 (1, 76.9654)	2.1822	-2.1306 to 6.1515	0.3242
		EBV	0.0890 (1, 100.3094)	0.6073	-3.2431 to 4.6126	0.7661
**C3**	133	TsinceIndex	0.0663 (1, 125.7775)	0.0041	-0.0256 to 0.0359	0.7973
		Comorbidity	0.3501 (1, 67.8980)	-0.0451	-0.1923 to 0.0981	0.556
		Vaccination	2.1032 (1, 69.7707)	0.0604	-0.0230 to 0.1489	0.1515
		Age	1.3327 (1, 78.8728)	0.0241	-0.0155 to 0.0661	0.2518
		Sex (M=1, F=0)	0.1940 (1, 76.9539)	0.0182	-0.0576 to 0.1026	0.6609
		EBV	0.0003 (1, 102.1705)	0.0007	-0.0765 to 0.0728	0.986
**C4**	133	TsinceIndex	0.9302 (1, 119.8654)	-0.0038	-0.0115 to 0.0040	0.3368
		Comorbidity	0.0132 (1, 67.2889)	-0.0024	-0.0410 to 0.0373	0.9088
		Vaccination	0.3544 (1, 70.1166)	0.0067	-0.0153 to 0.0277	0.5535
		Age	0.9285 (1, 82.1063)	0.0053	-0.0049 to 0.0159	0.3381
		Sex (M=1, F=0)	0.0115 (1, 78.5566)	0.0012	-0.0202 to 0.0215	0.9147
		EBV	0.3596 (1, 110.8580)	0.0059	-0.0128 to 0.0257	0.5499

b. SARS-CoV-2 related cytokines

Analyte	obs (n)	Predictor	F (df1, df2)	Estimate	95% CI	raw P
IFN-alpha	136	TsinceIndex	30.9665 (1, 102.9725)	-8.3157	-11.2389 to -5.5041	<0.0001
		Comorbidity	1.5207 (1, 59.8338)	-11.3253	-29.1557 to 5.7932	0.2223
		Vaccination	0.0532 (1, 59.4833)	-1.1925	-11.4569 to 9.2811	0.8184
		Age	1.3826 (1, 61.3045)	3.0644	-2.1685 to 8.3407	0.2442

Analyte	obs (n)	Predictor	F (df1, df2)	Estimate	95% CI	raw P
		Sex (M=1, F=0)	3.5246 (1, 59.0509)	9.8955	-0.5504 to 20.0692	0.0654
		EBV	4.2148 (1, 119.5967)	8.7201	0.7356 to 17.0552	**0.0423**
IL-6	136	TsinceIndex	13.5014 (1, 108.4902)	-10.2454	-15.8166 to -4.4073	0.0004
		Comorbidity	0.0455 (1, 64.9710)	-3.57	-35.8117 to 29.6540	0.8318
		Vaccination	0.5641 (1, 64.5790)	-7.0814	-26.1728 to 12.1061	0.4554
		Age	1.4500 (1, 66.4771)	5.7238	-3.7619 to 15.7567	0.2328
		Sex (M=1, F=0)	3.2903 (1, 64.1705)	17.4285	-0.7679 to 35.7653	0.0744
		EBV	3.6340 (1, 118.9562)	14.9338	-0.1224 to 29.7869	0.0590
IL-13	136	TsinceIndex	117.4069 (1, 68.7002)	-17.3693	-20.3142 to -14.3255	<0.0001
		Comorbidity	0.8535 (1, 64.5963)	-18.6482	-58.1815 to 18.6766	0.3590
		Vaccination	0.0575 (1, 64.7193)	2.7269	-19.0886 to 25.4233	0.8112
		Age	3.0718 (1, 65.1245)	9.9769	-0.8063 to 20.8195	0.0844
		Sex (M=1, F=0)	3.0299 (1, 64.2325)	20.2407	-2.1534 to 42.8667	0.0865
		EBV	0.5342 (1, 96.9720)	4.2162	-6.6451 to 15.1540	0.4666

c. Innate-associated cytokines

Analyte	obs (n)	Predictor	F (df1, df2)	Estimate	95% CI	raw P
IL-1alpha	136	TsinceIndex	7.6888 (1, 120.8035)	-2.8818	-5.1918 to -0.9054	0.0064
		Comorbidity	1.5327 (1, 61.9656)	-6.8186	-18.2807 to 4.2099	0.2204
		Vaccination	1.9113 (1, 61.3236)	-4.2843	-10.3878 to 1.7345	0.1718
		Age	0.5073 (1, 63.5930)	1.116	-1.6963 to 4.2778	0.4789
		Sex (M=1, F=0)	2.1687 (1, 61.1038)	4.6484	-1.8231 to 10.8158	0.1460
		EBV	7.7586 (1, 104.5994)	7.6022	2.4221 to 12.8164	0.0063
IL-1β	136	TsinceIndex	0.1188 (1, 92.7449)	0.3426	-1.4428 to 2.4099	0.7311
		Comorbidity	0.7581 (1, 65.3465)	-6.2087	-20.6272 to 7.8925	0.3871
		Vaccination	0.9042 (1, 65.2089)	-3.8197	-12.1567 to 4.0530	0.3452
		Age	0.9261 (1, 66.5921)	1.9431	-1.9111 to 5.6236	0.3394
		Sex (M=1, F=0)	4.1613 (1, 64.6493)	8.358	0.8431 to 16.1785	0.0454
		EBV	4.9895 (1, 128.6919)	6.7727	1.1255 to 12.9422	**0.0272**
GM-CSF	136	TsinceIndex	1.5054 (1, 105.1694)	-0.5996	-1.4708 to 0.3649	0.2226
		Comorbidity	0.7454 (1, 66.8187)	-2.6483	-8.7871 to 3.1248	0.3910
		Vaccination	0.0022 (1, 66.5008)	-0.0813	-3.7401 to 3.1774	0.9626
		Age	1.7027 (1, 68.2624)	1.1354	-0.6896 to 2.8915	0.1963
		Sex (M=1, F=0)	3.4213 (1, 66.0435)	3.2568	-0.0945 to 6.7086	0.0688
		EBV	4.0249 (1, 122.8621)	2.8157	0.0909 to 5.4269	0.0470
TNF-alpha	136	TsinceIndex	0.6789 (1, 116.2504)	62.6191	-96.1807 to 211.8993	0.4116
		Comorbidity	0.7576 (1, 67.9638)	-270.606	-872.2837 to 340.5532	0.3872
		Vaccination	0.0307 (1, 66.5121)	-30.5149	-394.6246 to 304.4905	0.8614
		Age	0.6550 (1, 69.1902)	72.0439	-99.1294 to 246.0072	0.4211
		Sex (M=1, F=0)	5.7729 (1, 67.0318)	426.4619	65.2576 to 781.8016	0.0191
		EBV	7.0555 (1, 81.8964)	449.8793	99.9196 to 750.3758	0.0095
IL-15	136	TsinceIndex	15.0291 (1, 125.9663)	-38.9436	-59.4189 to -20.3002	0.0002
		Comorbidity	1.4488 (1, 59.0952)	-60.3926	-158.5127 to 34.2072	0.2335
		Vaccination	1.2952 (1, 58.3144)	-32.1115	-88.2833 to 23.0183	0.2597
		Age	0.8648 (1, 60.7310)	13.2916	-16.2765 to 41.0003	0.3561
		Sex (M=1, F=0)	3.7595 (1, 58.2136)	55.7179	-1.0961 to 110.4158	0.0574
		EBV	8.5183 (1, 95.5353)	74.367	20.8550 to 122.7316	**0.0044**
IL-18	136	TsinceIndex	2.3730 (1, 94.5713)	-7.0125	-15.3152 to 1.9495	0.1268
		Comorbidity	0.3103 (1, 67.6355)	9.5835	-22.8161 to 43.4613	0.5793

Analyte	obs (n)	Predictor	F (df1, df2)	Estimate	95% CI	raw P
		Vaccination	0.6618 (1, 65.7913)	7.8074	-10.1133 to 25.2900	0.4188
		Age	1.7347 (1, 68.3066)	-6.5027	-16.3122 to 3.3653	0.1922
		Sex (M=1, F=0)	5.5901 (1, 66.7272)	23.168	3.6273 to 42.5224	0.0210
		EBV	4.2814 (1, 74.4913)	19.8455	0.8662 to 39.9876	**0.0420**

d.Th1/Th2 Balance (IL-4/IFNγ)

**Analyte**	**obs (n)**	**Predictor**	**F (df1, df2)**	**Estimate**	**95% CI**	**raw P**
IL-4 / IFNγ ratio	136	TsinceIndex	0.0492 (1, 116.6014)	0.0048	-0.0406 to 0.0486	0.8248
		Comorbidity	0.3641 (1, 66.3096)	0.054	-0.1154 to 0.2217	0.5483
		Vaccination	0.2045 (1, 64.8755)	0.0227	-0.0737 to 0.1214	0.6527
		Age	0.3032 (1, 67.5614)	0.0141	-0.0336 to 0.0686	0.5837
		Sex (M=1, F=0)	0.2634 (1, 65.3759)	-0.0262	-0.1264 to 0.0686	0.6095
		EBV	1.5071 (1, 80.7721)	-0.0598	-0.1475 to 0.0351	0.2231

e.Th17/22-associated cytokines

**Analyte**	obs (n)	Predictor	F (df1, df2)	Estimate	95% CI	raw P
IL-12p40	136	TsinceIndex	0.1997 (1, 122.2443)	51.1136	-170.3487 to 276.7515	0.6557
		Comorbidity	1.0906 (1, 66.0406)	-629.4195	-1799.5231 to 584.8103	0.3002
		Vaccination	0.4986 (1, 65.3846)	-239.4511	-897.3348 to 417.9618	0.4826
		Age	0.2428 (1, 67.6700)	84.5142	-251.8513 to 420.6164	0.6238
		Sex (M=1, F=0)	6.5154 (1, 65.1746)	881.675	236.2351 to 1592.6432	0.0130
		EBV	11.0355 (1, 106.4001)	994.594	415.1102 to 1568.7524	**0.0012**
IL-17A	136	TsinceIndex	0.2722 (1, 124.8386)	-0.0661	-0.3194 to 0.1860	0.6028
		Comorbidity	0.2876 (1, 67.3414)	-0.2934	-1.4294 to 0.7755	0.5935
		Vaccination	1.4324 (1, 66.1084)	-0.3674	-0.9452 to 0.2547	0.2356
		Age	0.0218 (1, 68.7851)	-0.0231	-0.3560 to 0.2662	0.8830
		Sex (M=1, F=0)	1.3595 (1, 66.4126)	0.3646	-0.2558 to 0.9710	0.2478
		EBV	3.2645 (1, 86.4924)	0.5294	-0.0376 to 1.1244	0.0743
IL-17F	136	TsinceIndex	3.4153 (1, 105.0353)	-1.8705	-3.9718 to -0.0158	0.0674
		Comorbidity	0.8539 (1, 66.7503)	-5.8766	-18.0163 to 6.3702	0.3588
		Vaccination	0.0342 (1, 66.4340)	0.662	-5.7798 to 7.9637	0.8539
		Age	1.4463 (1, 68.1928)	2.1695	-1.1854 to 5.9102	0.2333
		Sex (M=1, F=0)	4.4687 (1, 65.9756)	7.7169	0.1193 to 15.5337	0.0383
		EBV	3.8468 (1, 122.9180)	5.7041	0.1220 to 11.4545	0.0521
IL-22	136	TsinceIndex	10.0375 (1, 116.8028)	-2.1017	-3.4260 to -0.8201	0.0020
		Comorbidity	0.2455 (1, 64.9347)	-1.824	-8.3211 to 5.3329	0.6219
		Vaccination	0.0797 (1, 64.3917)	0.585	-3.5076 to 4.7117	0.7786
		Age	0.9407 (1, 66.5286)	1.0149	-0.8921 to 2.9798	0.3356
		Sex (M=1, F=0)	5.1208 (1, 64.0930)	4.7765	0.4507 to 9.0213	0.0270
		EBV	4.1788 (1, 111.3021)	3.6543	0.0342 to 7.1583	**0.0433**

f. Regulatory cytokines

**Analyte**	obs (n)	Predictor	F (df1, df2)	Estimate	95% CI	raw P
IL-11	136	TsinceIndex	24.0098 (1, 101.6221)	-7.1604	-10.0351 to -4.2351	<0.0001
		Comorbidity	1.8030 (1, 62.4370)	-12.4124	-32.1264 to 6.7767	0.1842
		Vaccination	0.4367 (1, 62.1314)	-3.4405	-13.4011 to 7.7214	0.5112
		Age	2.3446 (1, 63.8723)	4.0149	-1.1453 to 9.3585	0.1307
		Sex (M=1, F=0)	1.5238 (1, 61.6666)	6.5506	-3.7196 to 16.3599	0.2217
		EBV	6.5693 (1, 122.6172)	10.7974	2.5200 to 17.9975	**0.0116**

Analyte	obs (n)	Predictor	F (df1, df2)	Estimate	95% CI	raw P
IL-10	136	TsinceIndex	5.5922 (1, 124.6465)	-0.6048	-1.0728 to -0.1105	0.0196
		Comorbidity	0.2155 (1, 66.6375)	-0.6091	-3.1476 to 1.8004	0.6440
		Vaccination	0.1111 (1, 65.9183)	-0.246	-1.6054 to 1.1069	0.7400
		Age	1.3472 (1, 68.2758)	0.4336	-0.2981 to 1.1660	0.2498
		Sex (M=1, F=0)	3.9642 (1, 65.7602)	1.4966	-0.0937 to 2.9599	0.0506
		EBV	6.1582 (1, 103.8895)	1.6356	0.3592 to 2.8537	**0.0147**
IL-27	136	TsinceIndex	9.8878 (1, 84.0096)	-37.6827	-61.2812 to -15.1229	0.0023
		Comorbidity	0.8600 (1, 64.5455)	-90.1409	-293.8003 to 101.5830	0.3572
		Vaccination	0.8985 (1, 64.5236)	-51.911	-158.6306 to 45.8202	0.3467
		Age	0.8235 (1, 65.6047)	24.947	-26.3503 to 77.3958	0.3675
		Sex (M=1, F=0)	2.6664 (1, 63.9236)	91.2703	-27.7403 to 198.6589	0.1074
		EBV	4.8595 (1, 127.4497)	84.5259	9.3582 to 154.8435	**0.0293**

g. Autoantibodies

Analyte	obs (n)	Predictor	F (df1, df2)	Estimate	95% CI	raw P
Anti-Prothrombin	136	TsinceIndex	20.1861 (1, 109.0069)	-1.7558	-2.5502 to -0.9580	<0.0001
		Comorbidity	0.0014 (1, 67.5455)	0.089	-4.8482 to 4.6129	0.9701
		Vaccination	0.2838 (1, 67.1689)	0.7101	-1.9337 to 3.2386	0.5959
		Age	0.8251 (1, 69.0360)	0.6102	-0.6623 to 2.0265	0.3669
		Sex (M=1, F=0)	2.2601 (1, 66.7510)	-2.0418	-4.6059 to 0.5632	0.1375
		EBV	3.2898 (1, 120.3521)	2	-0.2226 to 4.2796	0.0722
Anti-CCP	136	TsinceIndex	0.7553 (1, 98.4492)	-0.3022	-0.9598 to 0.3871	0.3869
		Comorbidity	5.7271 (1, 67.9234)	5.6897	1.2613 to 10.3561	0.0195
		Vaccination	0.7368 (1, 67.7283)	1.1496	-1.5218 to 3.6657	0.3937
		Age	0.4190 (1, 69.2384)	0.436	-0.8210 to 1.7495	0.5196
		Sex (M=1, F=0)	1.5040 (1, 67.1988)	-1.6748	-4.3746 to 0.8563	0.2243
		EBV	0.0779 (1, 127.4979)	0.29	-1.7653 to 2.3843	0.7806
Anti-TransG	136	TsinceIndex	1.6355 (1, 74.9042)	0.3846	-0.2069 to 1.0107	0.2049
		Comorbidity	0.7110 (1, 66.6326)	-2.6865	-8.8466 to 3.3709	0.4021
		Vaccination	3.1556 (1, 66.7357)	3.1882	-0.2688 to 6.5978	0.0802
		Age	0.0587 (1, 67.3368)	-0.2179	-1.9283 to 1.4759	0.8093
		Sex (M=1, F=0)	0.0179 (1, 66.1736)	-0.2456	-3.5136 to 3.3990	0.8939
		EBV	1.3903 (1, 110.9859)	-1.2293	-3.2905 to 0.7120	0.2409
M3-mAChR AAK	137	TsinceIndex	0.6821 (1, 126.2062)	0.918	-1.2678 to 3.2187	0.4104
		Comorbidity	1.4000 (1, 67.2511)	5.7304	-4.1050 to 14.9778	0.2409
		Vaccination	0.5997 (1, 70.1476)	-2.0544	-7.5731 to 3.2095	0.4413
		Age	0.2943 (1, 77.2695)	0.7351	-1.9160 to 3.4406	0.5890
		Sex (M=1, F=0)	0.7065 (1, 76.8778)	2.2374	-3.5251 to 7.4931	0.4032
		EBV	8.3462 (1, 94.3314)	7.2379	1.9072 to 12.0500	0.0048
M4-mAChR AAK	137	TsinceIndex	1.8083 (1, 123.1557)	-1.1688	-2.7845 to 0.5206	0.1812
		Comorbidity	0.6186 (1, 66.2147)	3.6252	-5.3600 to 12.6264	0.4344
		Vaccination	0.0656 (1, 70.3677)	-0.6401	-5.4490 to 4.1419	0.7986
		Age	0.5322 (1, 83.2956)	0.9073	-1.5916 to 3.4968	0.4677
		Sex (M=1, F=0)	0.0201 (1, 80.0846)	0.3475	-4.7364 to 5.0171	0.8877
		EBV	6.5382 (1, 113.0733)	4.7318	1.1452 to 7.8124	0.0119

h. Granulocytes

Analyte	obs (n)	Predictor	F (df1, df2)	Estimate	95% CI	raw P
Neutrophils	137	TsinceIndex	0.8522 (1, 85.9325)	0.1015	-0.1179 to 0.3219	0.3585
		Comorbidity	2.0040 (1, 62.5503)	-0.5834	-1.3339 to 0.2628	0.1618
		Vaccination	10.5684 (1, 63.4130)	0.7415	0.3017 to 1.2319	0.0018
		Age	8.8916 (1, 66.4799)	0.3548	0.1190 to 0.5897	0.0040
		Sex (M=1, F=0)	0.2970 (1, 66.8155)	0.127	-0.3244 to 0.5933	0.5876
		EBV	5.4935 (1, 71.8285)	0.5331	0.0775 to 0.9657	**0.0219**
Eosinophils	137	TsinceIndex	3.3267 (1, 114.5374)	0.0255	-0.0027 to 0.0531	0.0708
		Comorbidity	0.0710 (1, 66.2732)	0.0152	-0.0962 to 0.1320	0.7908
		Vaccination	0.7027 (1, 68.6584)	-0.0264	-0.0881 to 0.0340	0.4048
		Age	9.0526 (1, 74.0629)	-0.0489	-0.0798 to -0.0183	0.0036
		Sex (M=1, F=0)	0.7077 (1, 73.8811)	0.0268	-0.0292 to 0.0906	0.4029
		EBV	0.0025 (1, 85.8172)	-0.0015	-0.0623 to 0.0540	0.9604
Basophils	137	TsinceIndex	0.4621 (1, 128.3612)	0.0012	-0.0024 to 0.0045	0.4979
		Comorbidity	0.0716 (1, 67.4922)	-0.002	-0.0170 to 0.0119	0.7899
		Vaccination	4.2184 (1, 70.9290)	0.0085	-0.0002 to 0.0165	0.0437
		Age	0.0654 (1, 78.9578)	-0.0005	-0.0047 to 0.0039	0.7988
		Sex (M=1, F=0)	0.4704 (1, 77.8546)	-0.0029	-0.0113 to 0.0056	0.4948
		EBV	0.0067 (1, 97.0784)	-0.0003	-0.0079 to 0.0068	0.9351

i. Mental Health

Analyte	obs (n)	Predictor	F (df1, df2)	Estimate	95% CI	raw P
SF-12 PCS	137	TsinceIndex	0.4621 (1, 128.3612)	0.0012	-0.0024 to 0.0045	0.4979
		Comorbidity	0.0716 (1, 67.4922)	-0.002	-0.0170 to 0.0119	0.7899
		Vaccination	4.2184 (1, 70.9290)	0.0085	-0.0002 to 0.0165	0.0437
		Age	0.0654 (1, 78.9578)	-0.0005	-0.0047 to 0.0039	0.7988
		Sex (M=1, F=0)	0.4704 (1, 77.8546)	-0.0029	-0.0113 to 0.0056	0.4948
		EBV	0.0067 (1, 97.0784)	-0.0003	-0.0079 to 0.0068	0.9351
PHQ-9	138	TsinceIndex	11.5632 (1, 128.1644)	-1.6381	-2.5975 to -0.7016	0.0009
		Comorbidity	0.2100 (1, 65.8572)	0.9767	-3.2592 to 5.5588	0.6483
		Vaccination	0.3915 (1, 68.7232)	-0.7298	-3.0585 to 1.7432	0.5336
		Age	1.3805 (1, 76.0060)	0.6987	-0.3869 to 1.8441	0.2437
		Sex (M=1, F=0)	0.3534 (1, 75.2205)	-0.694	-2.9546 to 1.6545	0.5540
		EBV	0.9549 (1, 94.0475)	1.0709	-1.0301 to 3.3019	0.3310
GAD-7	138	TsinceIndex	5.3317 (1, 128.9848)	-0.9675	-1.8107 to -0.1759	0.0225
		Comorbidity	0.0804 (1, 66.0600)	0.5305	-2.9041 to 4.1941	0.7777
		Vaccination	0.0621 (1, 69.0227)	0.2551	-1.6112 to 2.2032	0.8040
		Age	0.2566 (1, 76.5817)	0.2641	-0.7210 to 1.3336	0.6139
		Sex (M=1, F=0)	0.1677 (1, 75.6958)	-0.4192	-2.2688 to 1.4859	0.6833
		EBV	2.2725 (1, 95.2333)	1.5834	-0.2178 to 3.9955	0.1350

Supplementary Table 5a | LMM screening of 43 metabolic and biomarker parameters. Related to Table 3. Two-sided linear mixed-effects models (LMMs) were fitted separately for each of 43 blood-based clinical laboratory parameters, with participant ID included as a random intercept. Fixed effects comprised TsinceIndex (LC ≤1 year versus LC 1–3 yr), anti-EBV EBNA serostatus, anti-DFS70 aAb status, and all two-way and three-way interaction terms. P values were estimated using Satterthwaite's approximation and validated by non-parametric percentile bootstrap (1,000 iterations). Raw and Holm–Bonferroni-adjusted P values are reported across all 43 parameters; adjusted $P < 0.05$ was considered significant. R^2m indicates the variance explained by the fixed

effects. Effect-specific P values for the main effects and interaction terms are shown only for analytes whose overall model remained significant after Holm–Bonferroni correction; corresponding cells for all other analytes were left blank by design. Full effect-level summaries across all 43 analytes are provided in Supplementary Table 5b. Abbreviations: aAb, autoantibody; ACTH, adrenocorticotropic hormone; ALT, alanine aminotransferase; aPTT, activated partial thromboplastin time; AST, aspartate aminotransferase; CK, creatine kinase; CK-MB, creatine kinase myocardial band; DFS70, dense fine speckled 70; EBV, Epstein–Barr virus; eGFR, estimated glomerular filtration rate; hs-cTnT, high-sensitivity cardiac troponin T; hs-CRP, high-sensitivity C-reactive protein; IgA, immunoglobulin A; IgG, immunoglobulin G; IgM, immunoglobulin M; LC, long COVID; LDH, lactate dehydrogenase; LMM, linear mixed-effects model; Lp(a), lipoprotein(a); obs, observations; RDW, red cell distribution width; TSH, thyroid-stimulating hormone.

Analyte	P	obs (n)	R^2_{Mag}	**Adj. P**	Time (T)	EBV (E)	DFS70 (D)	Interaction T*D	Interaction E*D	Interaction T*E	Interaction T*E*D
TSH	**<.0001**	137	0.4141	**0.0043**	<.0001	<.0001	<.0001	<.00001	<.0001	<.0001	<.0001
aPTT	**<.0001**	138	0.2477	**0.0043**	0.0004	0.0025	0.0056	0.0003	0.051	0.2716	0.0003
Lp(a)	**0.0003**	131	0.1816	**0.0123**	0.0015	0.0753	0.0078	0.0022	0.0146	0.0053	0.0022
RDW	**0.0016**	137	0.1636	0.064							
IgA	**0.0018**	138	0.1875	0.0702							
Vitamin D	**0.0039**	136	0.1721	0.1482							
CRP HS mg/l	0.0095	111	0.1379	0.3515							
Factor VIII	0.0171	138	0.1522	0.6156							
Zinc	0.0545	137	0.1071	1.0000							
Selenium	0.0591	136	0.0942	1.0000		**Holm–Bonferroni Adj. P ≥ 0.05**					
Bicarbonate	0.0635	127	0.0941	1.0000							
Chloride	0.0644	137	0.0935	1.0000							
Holotranscobalamin	0.1731	73	0.1339	1.0000							
Creatinine	0.2347	137	0.0875	1.0000							
Vitamin B12	0.257	133	0.0466	1.0000							
Lipase	0.2655	137	0.0517	1.0000							
LDH	0.2696	137	0.0625	1.0000							
D-dimer	0.2702	138	0.0591	1.0000							
Fibrinogen	0.3118	138	0.0604	1.0000							
Potassium	0.3141	137	0.0677	1.0000							
Free proteinS	0.3221	137	0.0611	1.0000							
Ferritin	0.3262	137	0.0475	1.0000							
CK	0.4264	137	0.0531	1.0000							
Vitamin B6	0.4592	136	0.0577	1.0000		**Holm–Bonferroni Adj. P ≥ 0.05**					
Folate	0.4755	137	0.0364	1.0000							
Glucose	0.511	127	0.0455	1.0000							
Lactate	0.5453	131	0.0438	1.0000							
Phosphate	0.55	137	0.0478	1.0000							
hs-cTnT	0.5537	137	0.0474	1.0000							
IgM	0.5729	138	0.0366	1.0000							
IgG	0.5746	138	0.0353	1.0000							
CK-MB	0.6354	137	0.0361	1.0000							
Oxygen	0.6761	127	0.0381	1.0000							
ACTH	0.7157	137	0.0416	1.0000							
AST	0.7509	137	0.0299	1.0000							
Blood pH	0.7893	127	0.0314	1.0000							
Calcium	0.8242	137	0.0278	1.0000							
Urine pH	0.8628	127	0.0251	1.0000							
Triglycerides	0.8764	135	0.0243	1.0000							
ALT	0.908	137	0.0131	1.0000							
eGFR	0.9322	138	0.0166	1.0000							
Vitamin B1	0.9594	134	0.0171	1.0000							
Uric acid	0.9776	111	0.0119	1.0000							

Supplementary Table 5b | Effect-level model output for the three parameters remaining significant after multiple-testing correction, plus effect-specific summaries across all 43 parameters. Related to Table 3. Two-sided linear mixed-effects models (LMMs) are shown for the three parameters that remained significant after Holm–Bonferroni adjustment across 43 tested parameters. Models included TsinceIndex as a binary variable (LC ≤1 year versus LC 1–3 yr), anti-EBV EBNA status,

anti-DFS70 aAb status, and all relevant two-way and three-way interaction terms, with participant ID included as a random intercept. For each fixed effect, F statistics with numerator and residual degrees of freedom, parameter estimates, 95% confidence intervals, and raw P values are reported. Degrees of freedom were estimated using Satterthwaite's approximation, and 95% confidence intervals were obtained by percentile bootstrap (1,000 resamples). Model fit showed marginal and conditional R^2 values of 0.1816 and 0.9416 for LP(a), 0.2477 and 0.7023 for aPTT, and 0.4141 and 0.6991 for TSH, respectively; all models were significant by likelihood-ratio testing (all $P < 0.0001$, except LP(a), $P = 0.0003$). Lower panels summarize the fixed effects of TsinceIndex, anti-DFS70 status, and anti-EBV EBNA status across all 43 analytes.

LP(a)

Predictor	F (df1, df2)	Estimate	95% CI	raw P
TsinceIndex	10.8515 (1, 70.7058)	-90.5793	-140.6564 to -37.9835	0.0015
DFS70	7.5063 (1, 69.4132)	77.8142	19.9763 to 130.9512	0.0078
EBV	3.2562 (1, 72.0769)	50.2246	-8.0446 to 104.6886	0.0753
TsinceIndex × DFS70	10.1337 (1, 70.7058)	-175.0643	-277.2893 to -72.6404	0.0022
TsinceIndex × EBV	8.2854 (1, 70.9029)	-158.4385	-268.2688 to -51.7122	0.0053
DFS70 × EBV	6.2645 (1, 72.0769)	139.3277	26.2671 to 251.1839	0.0146
TsinceIndex × DFS70 × EBV	7.9643 (1, 70.9029)	-310.6771	-527.0259 to -99.8924	0.0062

aPTT

Predictor	F (df1, df2)	Estimate	95% CI	raw P
TsinceIndex	13.3700 (1, 83.8713)	-3.9609	-6.0473 to -1.7776	0.0004
DFS70	8.1085 (1, 79.6957)	3.1320	0.9880 to 5.2495	0.0056
EBV	9.7016 (1, 81.4417)	3.4109	1.3755 to 5.4691	0.0025
TsinceIndex × DFS70	14.3897 (1, 83.8713)	-8.2183	-12.5283 to -3.6742	0.0003
TsinceIndex × EBV	1.2246 (1, 83.7277)	-2.4016	-6.5191 to 2.1265	0.2716
DFS70 × EBV	3.9234 (1, 81.4417)	4.3382	0.1020 to 8.6903	0.0510
TsinceIndex × DFS70 × EBV	2.2544 (1, 83.7277)	-6.5169	-15.0383 to 2.4404	0.1370

TSH

Predictor	F (df1, df2)	Estimate	95% CI	raw P
TsinceIndex	23.0038 (1, 93.0667)	2.0647	1.2128 to 2.9165	<0.0001
DFS70	40.0124 (1, 88.8778)	2.7532	1.8919 to 3.6145	<0.0001
EBV	23.6385 (1, 90.2590)	2.1102	1.2514 to 2.9691	<0.0001
TsinceIndex × DFS70	28.6769 (1, 93.0667)	4.6105	2.9068 to 6.3141	<0.0001
TsinceIndex × EBV	30.3013 (1, 92.7434)	4.7462	3.0401 to 6.4524	<0.0001
DFS70 × EBV	25.1040 (1, 90.2590)	4.3493	2.6316 to 6.0671	<0.0001
TsinceIndex × DFS70 × EBV	25.9706 (1, 92.7434)	8.7880	5.3756 to 12.2003	<0.0001

TsinceIndex effect across all 43 analytes

Analyte	F (df1, df2)	Estimate	95% CI lower to upper	raw P
TSH	23.0038 (1, 93.0667)	2.0647	1.1913 to 2.8685	<.0001
aPTT	13.3700 (1, 83.8713)	-3.9609	-6.1676 to -1.7687	0.0004
Lp(a)	10.8515 (1, 70.7058)	-90.5793	-143.8749 to -35.9046	0.0015
ACTH	0.0472 (1, 84.9612)	-1.1777	-11.6833 to 9.7447	0.8285
ALT	0.0814 (1, 71.7088)	0.0516	-0.3037 to 0.3812	0.7763
AST	0.4251 (1, 77.8481)	-0.0714	-0.2833 to 0.1597	0.5163

Analyte	F (df1, df2)	Estimate	95% CI lower to upper	raw P
Blood pH	0.0320 (1, 91.5232)	0.0020	-0.0189 to 0.0256	0.8585
CK	0.0051 (1, 115.2715)	-0.1037	-2.9407 to 2.7649	0.9433
CK-MB	0.2850 (1, 79.4893)	-0.2475	-1.1130 to 0.7443	0.5949
Calcium	0.1368 (1, 101.9214)	-0.0131	-0.0835 to 0.0567	0.7122
Chloride	0.8824 (1, 111.7689)	0.7254	-0.7760 to 2.2398	0.3496
Creatinine	0.0001 (1, 78.6462)	0.0683	-9.9409 to 11.3401	0.9903
D-dimer	0.2316 (1, 120.1178)	19.4825	-58.8297 to 99.7768	0.6312
Factor VIII	2.3492 (1, 92.0462)	18.7727	-5.5424 to 42.2800	0.1288
Ferritin	0.3004 (1, 76.8874)	-10.4783	-48.4507 to 27.8592	0.5852
Fibrinogen	0.9793 (1, 77.5560)	0.2168	-0.2136 to 0.6296	0.3254
Folate	0.2752 (1, 74.4616)	-1.5865	-7.2925 to 4.1967	0.6014
Free protein S	5.3227 (1, 81.5742)	-18.5697	-35.3476 to -2.8059	0.0236
Glucose	0.4001 (1, 119.0000)	0.1652	-0.3773 to 0.6786	0.5283
Holotranscobalamin	1.1657 (1.0000, 43.0867)	15.0760	-11.1751 to 38.6771	0.2863
IgA	0.8850 (1, 105.6613)	-0.5419	-1.6253 to 0.5437	0.3490
IgG	1.0169 (1, 73.4721)	0.9679	-0.9109 to 2.8971	0.3166
IgM	0.5230 (1, 70.5523)	0.1474	-0.2331 to 0.5689	0.4720
LDH	3.2929 (1, 86.2536)	-0.4948	-0.9999 to 0.0234	0.0731
Lactate	0.3159 (1, 109.8061)	-0.0904	-0.4223 to 0.2079	0.5752
Lipase	0.0650 (1, 74.0014)	-0.0153	-0.1289 to 0.0888	0.7994
Oxygen	0.1442 (1, 101.2408)	-0.3562	-2.2876 to 1.4061	0.7049
Phosphate	0.9725 (1, 84.2751)	-0.0908	-0.2675 to 0.0933	0.3269
Potassium	0.0164 (1, 92.9724)	0.0140	-0.2028 to 0.2182	0.8983
RDW	9.8620 (1, 76.3516)	0.8855	0.2936 to 1.4722	0.0024
Selenium	0.1909 (1, 84.0881)	-0.0263	-0.1480 to 0.0897	0.6633
Standard bicarbonate	0.5950 (1, 85.7801)	0.3418	-0.4564 to 1.1522	0.4426
Triglycerides	0.3641 (1, 100.5375)	-0.1473	-0.5973 to 0.3396	0.5476
Uric acid	0.5203 (1.0000, 64.8662)	26.3469	-44.9953 to 92.4289	0.4733
Urine pH	0.0048 (1, 93.0272)	-0.0293	-0.8727 to 0.8179	0.9447
Vitamin B1	0.3248 (1, 94.9403)	8.4384	-21.1339 to 38.5476	0.5701
Vitamin B12	0.0096 (1, 79.4277)	-12.2694	-249.7698 to 231.7737	0.9220
Vitamin B6	0.3450 (1, 95.9158)	-27.7940	-118.2831 to 63.0868	0.5583
Vitamin D	5.5840 (1, 104.3312)	-30.1484	-54.6478 to -4.7638	0.0200
Zinc	0.2454 (1, 99.8496)	0.4402	-1.3016 to 2.2542	0.6215
eGFR	0.0937 (1, 84.4070)	2.0194	-12.0537 to 14.2052	0.7602
hs-CRP	0.0694 (1.0000, 62.7000)	0.0222	-0.8981 to 0.9363	0.7930
hs-cTnT	0.0001 (1, 106.0523)	-0.0083	-1.6178 to 1.6876	0.9921

Anti-DFS70 effect across all 43 analytes

Analyte	F (df1, df2)	Estimate	95% CI lower to upper	raw P
TSH	40.0124 (1, 88.8778)	2.7532	1.8972 to 3.6084	<0.0001
aPTT	8.1085 (1, 79.6957)	3.1320	1.0089 to 5.2095	0.0056
Lp(a)	7.5063 (1, 69.4132)	77.8142	22.8854 to 129.7002	0.0078

Analyte	F (df1, df2)	Estimate	95% CI lower to upper	raw P
ACTH	0.7416 (1, 80.7035)	-4.7334	-16.0995 to 6.6062	0.3917
ALT	0.0549 (1, 69.4927)	-0.0436	-0.4118 to 0.3154	0.8154
AST	0.0185 (1, 74.2611)	-0.0152	-0.2310 to 0.2086	0.8923
Blood pH	0.0190 (1, 87.6316)	0.0016	-0.0220 to 0.0241	0.8908
CK	0.1140 (1, 112.7367)	-0.4919	-3.4540 to 2.1155	0.7363
CK-MB	0.1500 (1, 76.0751)	0.1834	-0.7273 to 1.1735	0.6996
Calcium	0.0022 (1, 98.0940)	0.0017	-0.0654 to 0.0708	0.9623
Chloride	1.1476 (1, 108.7816)	-0.8292	-2.3447 to 0.5624	0.2864
Creatinine	3.9026 (1, 75.5412)	-11.3212	-22.1574 to -0.4652	0.0519
D-dimer	0.2982 (1, 118.1216)	-22.1102	-100.5071 to 52.0413	0.5860
Factor VIII	4.4219 (1, 87.8222)	-26.0576	-49.7055 to -4.4284	0.0383
Ferritin	1.0882 (1, 74.1216)	-20.4442	-58.3147 to 18.2591	0.3003
Fibrinogen	0.0401 (1, 73.9674)	-0.0448	-0.4546 to 0.3930	0.8419
Folate	0.0273 (1, 71.6603)	0.5119	-5.3712 to 6.3992	0.8693
Free protein S	1.2296 (1, 77.9114)	-9.0976	-24.9615 to 6.9686	0.2709
Glucose	1.6154 (1, 119.0000)	-0.3320	-0.8280 to 0.1832	0.2062
Holotranscobalamin	6.0054 (1.0000, 50.1273)	-37.1303	-64.5938 to -10.5638	0.0178
IgA	1.7587 (1, 102.0481)	0.7682	-0.3961 to 1.9107	0.1877
IgG	0.1254 (1, 71.5830)	0.3500	-1.5481 to 2.2409	0.7242
IgM	1.1236 (1, 69.5196)	0.2233	-0.1992 to 0.6351	0.2928
LDH	0.5093 (1, 82.0020)	0.1973	-0.3395 to 0.7434	0.4775
Lactate	0.9219 (1, 107.4702)	-0.1546	-0.4683 to 0.1741	0.3391
Lipase	0.0400 (1, 71.4106)	-0.0124	-0.1294 to 0.1156	0.8420
Oxygen	0.1319 (1, 98.0970)	-0.3414	-2.2508 to 1.5842	0.7173
Phosphate	2.4912 (1, 80.4493)	0.1480	-0.0129 to 0.3209	0.1184
Potassium	1.2534 (1, 88.8066)	0.1233	-0.1182 to 0.3346	0.2659
RDW	5.1704 (1, 73.3183)	0.6563	0.1302 to 1.2562	0.0259
Selenium	0.7941 (1, 80.0757)	0.0546	-0.0556 to 0.1723	0.3755
Standard bicarbonate	1.8998 (1, 81.9510)	0.6185	-0.1689 to 1.4937	0.1719
Triglycerides	0.0002 (1, 96.6661)	-0.0033	-0.5007 to 0.4748	0.9892
Uric acid	0.0029 (1.0000, 68.0557)	-9.6728	-81.4099 to 62.6226	0.9575
Urine pH	0.0114 (1, 89.0717)	0.0453	-0.7236 to 0.8399	0.9153
Vitamin B1	0.0005 (1, 91.0869)	0.3310	-28.6974 to 27.1305	0.9823
Vitamin B12	0.0368 (1, 76.2819)	24.4868	-225.0885 to 282.6802	0.8484
Vitamin B6	0.2913 (1, 92.1420)	-25.7481	-111.4605 to 71.9683	0.5907
Vitamin D	0.3105 (1, 100.7160)	7.1494	-19.5092 to 31.7942	0.5786
Zinc	0.2824 (1, 95.7632)	0.4753	-1.2775 to 2.2847	0.5964
eGFR	0.6003 (1, 80.5674)	-5.2003	-17.5935 to 7.3043	0.4407
hs-CRP	6.6581 (1.0000, 85.1250)	1.6469	0.5441 to 2.7155	0.0116
hs-cTnT	1.6800 (1, 102.4996)	-1.0951	-2.7431 to 0.4993	0.1978

Anti-EBV EBNA effect across all 43 analytes

Analyte	F (df1, df2)	Estimate	95% CI lower to upper	raw P
TSH	23.6385 (1, 90.2590)	2.1102	1.2610 to 2.9207	<0.0001
aPTT	9.7016 (1, 81.4417)	3.4109	1.2863 to 5.5022	0.0025
Lp(a)	3.2562 (1, 72.0769)	50.2246	-0.7104 to 105.1451	0.0753
ACTH	0.2702 (1, 82.3635)	-2.8460	-13.3096 to 8.4591	0.6046
ALT	0.0879 (1, 72.1790)	0.0544	-0.3138 to 0.4227	0.7677
AST	0.4959 (1, 76.3805)	0.0781	-0.1439 to 0.2707	0.4834
Blood pH	0.4264 (1, 88.5215)	-0.0075	-0.0308 to 0.0131	0.5155
CK	0.4701 (1, 113.2732)	0.9987	-1.8696 to 3.8103	0.4943
CK-MB	1.8194 (1, 78.2270)	0.6339	-0.2874 to 1.4917	0.1813
Calcium	0.0386 (1, 99.1273)	-0.0070	-0.0708 to 0.0635	0.8446
Chloride	4.0421 (1, 109.4381)	-1.5554	-3.0113 to -0.0702	0.0468
Creatinine	0.0006 (1, 77.8299)	0.1410	-10.7770 to 12.3390	0.9803
D-dimer	0.3859 (1, 118.5217)	25.1489	-55.6401 to 106.0314	0.5357
Factor VIII	0.0756 (1, 89.2583)	-3.3959	-27.0752 to 20.7620	0.7840
Ferritin	0.0434 (1, 76.5686)	4.0432	-32.8657 to 42.9783	0.8354
Fibrinogen	2.6211 (1, 76.0916)	0.3594	-0.0805 to 0.8070	0.1096
Folate	0.1976 (1, 74.1388)	1.3632	-4.5033 to 7.0192	0.6580
Free protein S	0.0365 (1, 79.9028)	1.5567	-14.5745 to 17.9132	0.8491
Glucose	2.7900 (1, 119.0000)	-0.4364	-0.9276 to 0.0847	0.0975
Holotranscobalamin	2.0638 (1.0000, 47.7606)	-24.7643	-56.4857 to 8.7000	0.1574
IgA	0.6447 (1, 102.9670)	-0.4645	-1.6246 to 0.6318	0.4239
IgG	0.2144 (1, 74.3096)	0.4508	-1.4742 to 2.3131	0.6447
IgM	3.0582 (1, 72.0825)	0.3604	-0.0409 to 0.7701	0.0846
LDH	3.4870 (1, 83.6339)	0.5143	-0.0342 to 1.0657	0.0654
Lactate	1.2338 (1, 107.9070)	-0.1788	-0.4856 to 0.1654	0.2691
Lipase	0.4533 (1, 73.9627)	-0.0411	-0.1553 to 0.0737	0.5029
Oxygen	1.7183 (1, 98.7227)	-1.2317	-3.1884 to 0.6528	0.1930
Phosphate	0.0001 (1, 82.3127)	-0.0008	-0.1819 to 0.1761	0.9935
Potassium	0.0344 (1, 90.2013)	0.0204	-0.2012 to 0.2254	0.8533
RDW	1.6961 (1, 75.6758)	0.3724	-0.2064 to 0.9920	0.1967
Selenium	1.7191 (1, 81.8357)	0.0799	-0.0407 to 0.1958	0.1935
Standard bicarbonate	1.6346 (1, 83.0570)	0.5721	-0.2749 to 1.4308	0.2046
Triglycerides	0.6313 (1, 97.6918)	0.1950	-0.2583 to 0.6711	0.4288
Uric acid	0.2650 (1.0000, 67.7699)	-26.3601	-117.0929 to 73.9953	0.6084
Urine pH	0.2256 (1, 90.1663)	0.2012	-0.6716 to 1.0217	0.6360
Vitamin B1	0.0754 (1, 92.2604)	-4.0892	-34.6370 to 24.8809	0.7843
Vitamin B12	0.0139 (1, 78.4484)	14.9343	-223.7406 to 259.8407	0.9064
Vitamin B6	0.3188 (1, 93.3667)	-26.8751	-123.6076 to 69.9630	0.5737
Vitamin D	0.2076 (1, 101.6375)	-5.8396	-30.9824 to 22.0119	0.6496
Zinc	0.7783 (1, 96.8353)	0.7878	-0.9024 to 2.5863	0.3799
eGFR	0.3693 (1, 82.4299)	-4.0572	-16.6607 to 9.0015	0.5450
hs-CRP	10.4840 (1.0000, 79.0623)	2.1774	0.8598 to 3.4374	0.0018
hs-cTnT	0.2300 (1, 103.3637)	0.4048	-1.2588 to 2.0551	0.6325

Supplementary Table 5c | Extended age- and sex-adjusted linear mixed-effects models for the three significant metabolic parameters. Related to Table 3. Two-sided linear mixed-effects models (LMMs) are shown for the three parameters that remained significant after Holm–Bonferroni adjustment across 43 tested parameters and were subsequently adjusted for age and sex. Models included TsinceIndex as a binary variable (LC ≤1 year versus LC 1–3 yr), anti-EBV EBNA status, anti-DFS70 aAb status, age, sex, and all relevant two-way and three-way interaction terms, with participant ID included as a random intercept. For each fixed effect, F statistics with numerator and residual degrees of freedom, parameter estimates, 95% confidence intervals, and raw P values are reported. Degrees of freedom were approximated using Satterthwaite's method, and 95% confidence intervals were obtained by percentile bootstrap (1,000 resamples). Model fit showed marginal and conditional R^2 values of 0.1974 and 0.9483 for LP(a), 0.4103 and 0.7030 for TSH, and 0.3315 and 0.7010 for PTT, respectively; all models were significant by likelihood-ratio testing (all P < 0.0001, except LP(a), P = 0.0001).

LP(a)

Predictor	F (df1, df2)	Estimate	95% CI	P
TsinceIndex	9.7488 (1, 68.5364)	-88.1301	-147.2162 to -31.4292	0.0026
DFS70	4.7260 (1, 69.7187)	63.8682	2.5483 to 119.9772	0.0331
EBV	3.3634 (1, 69.2425)	51.8133	-2.5382 to 102.6204	0.0710
Age	0.7332 (1, 104.4184)	-5.5298	-18.8421 to 6.9046	0.3938
Sex (M=1, F=0)	4.4148 (1, 93.5943)	-26.8969	-51.0523 to -2.6589	0.0383
TsinceIndex × DFS70	9.6389 (1, 68.1568)	-174.8017	-288.5638 to -63.1781	0.0028
TsinceIndex × EBV	6.6270 (1, 69.6622)	-144.8688	-261.4848 to -34.2957	0.0122
DFS70 × EBV	5.3457 (1, 68.9234)	130.1968	26.2134 to 233.3846	0.0238
TsinceIndex × DFS70 × EBV	6.5638 (1, 68.9242)	-287.6749	-531.5525 to -64.2164	0.0126

TSH

Predictor	F (df1, df2)	Estimate	95% CI	P
TsinceIndex	21.9692 (1, 89.0897)	2.0740	1.1492 to 2.8758	<0.0001
DFS70	37.0779 (1, 85.9752)	2.7892	1.9579 to 3.7230	<0.0001
EBV	23.3001 (1, 87.1400)	2.1272	1.3099 to 2.9694	<0.0001
Age	0.0016 (1, 78.8126)	-0.0046	-0.2287 to 0.2121	0.9681
Sex (M=1, F=0)	0.2557 (1, 77.1997)	0.1099	-0.3189 to 0.5389	0.6145
TsinceIndex × DFS70	27.6908 (1, 89.6669)	4.6446	2.8629 to 6.3048	<0.0001
TsinceIndex × EBV	27.7979 (1, 91.1534)	4.7208	2.9137 to 6.4059	<0.0001
DFS70 × EBV	24.6561 (1, 87.4050)	4.3589	2.7259 to 6.0689	<0.0001
TsinceIndex × DFS70 × EBV	24.8342 (1, 89.1156)	8.7834	5.2275 to 12.0876	<0.0001

PTT

Predictor	F (df1, df2)	Estimate	95% CI	P
TsinceIndex	11.5179 (1, 82.4730)	-3.5271	-5.6399 to -1.5209	0.0011
DFS70	8.8946 (1, 79.5097)	3.2125	1.2692 to 5.1778	0.0038
EBV	13.0787 (1, 80.3806)	3.7457	1.8334 to 5.7379	0.0005
Age	2.9309 (1, 74.8700)	-0.4572	-0.9847 to 0.0670	0.0910
Sex (M=1, F=0)	5.8391 (1, 74.3584)	1.2390	0.2853 to 2.2249	0.0181
TsinceIndex × DFS70	12.3303 (1, 83.0105)	-7.2777	-11.4968 to -3.3310	0.0007
TsinceIndex × EBV	0.8817 (1, 84.3503)	-1.9693	-6.0649 to 2.0775	0.3504
DFS70 × EBV	4.8845 (1, 80.5413)	4.5576	0.6375 to 8.3423	0.0299
TsinceIndex × DFS70 × EBV	2.1056 (1, 82.1251)	-5.9978	-14.3958 to 1.9957	0.1506

Supplementary Table 6a | Linear mixed-effects model (LMM) of Bell score in the EBV-naïve/low paediatric LC subgroup. Related to Fig. 4. A two-sided LMM was applied to the anti-EBV EBNA-negative/low subgroup of paediatric LC patients to assess associations of Bell score with IL-12p40, MCHC, absolute basophil granulocyte counts and vitamin B1, with participant ID included as a random intercept. Model fit shows marginal and conditional R^2 (R^2m = 0.318, R^2c = 0.853);

model significance was confirmed by likelihood-ratio testing (P < 0.0001). Degrees of freedom were approximated using Satterthwaite's method, and confidence intervals were derived by percentile bootstrap (1,000 resamples). No multiple-comparison adjustment was applied.

Bell score

Predictor	F (1, df2)	Estimate	95% CI	P
IL-12p40	6.8321 (1, 35.5107)	3.7157	0.9739 to 6.6601	0.0130
MCHC	12.4870 (1, 62.7262)	-8.3943	-12.9761 to -3.4395	0.0008
Baso. Granul.	6.0902 (1, 56.6442)	4.7450	0.9319 to 8.7520	0.0166
Vitamin B1	8.0014 (1, 50.8157)	5.9728	1.7207 to 10.2134	0.0067

Supplementary Table 6b | Confounder-adjusted Bell score model in the EBV-naïve/low paediatric LC subgroup. Related to Fig. 4. A two-sided linear mixed-effects model (LMM) was applied to the anti-EBV EBNA-negative/low subgroup of paediatric LC patients to assess associations of Bell score with IL-12p40, MCHC, absolute basophil granulocyte counts and vitamin B1 after adjustment for vaccination status, comorbidity, age, sex and TsinceIndex, with participant ID included as a random intercept. Model fit showed marginal and conditional R^2 values of 0.3169 and 0.8562, respectively, and was significant by likelihood-ratio testing (P < 0.0001). Degrees of freedom were approximated using Satterthwaite's method, and confidence intervals were derived by percentile bootstrap (1,000 resamples). No multiple-comparison adjustment was applied.

Bell score

Predictor	F (1, df2)	Estimate	95% CI	P
Comorbidity	0.0445 (1, 34.2564)	2.8304	-24.0452 to 29.7060	0.8342
Sex (M=1, F=0)	0.0931 (1, 39.6282)	-1.8934	-14.3175 to 10.5307	0.7618
Age	1.9037 (1, 38.5456)	4.1271	-1.8626 to 10.1167	0.1756
IL-12p40	7.9696 (1, 32.4347)	4.1284	1.2000 to 7.0568	0.0081
MCHC	10.5361 (1, 58.9073)	-8.0269	-12.9788 to -3.0750	0.0019
Baso. Granul.	5.7484 (1, 54.4877)	4.7005	0.7746 to 8.6263	0.0200
Vitamin B1	7.4327 (1, 47.2095)	5.9910	1.5906 to 10.3914	0.0090
TsinceIndex	0.1406 (1, 51.1670)	-0.7568	-4.7985 to 3.2849	0.7092

Supplementary Table 7 | Pre-existing conditions in paediatric LC: cohort prevalence versus reported population benchmarks. Pre-existing conditions recorded at enrolment among participants with paediatric LC (n = 74). Data are shown as n (%) unless stated otherwise. Published prevalence estimates are provided for contextual comparison only and should be interpreted with caution given heterogeneity in age strata, case definitions, data sources (population-based surveys vs clinical cohorts), and ascertainment; they were not used for statistical inference in this study. Benchmark estimates were obtained from population-based German health surveys, international systematic reviews, and disease-specific registries (e.g., Robert Koch Institute; KiGGS Wave 2, 2014–2017, published 2018). Participants could report more than one pre-existing condition; therefore, counts across conditions are not mutually exclusive and may sum to > 74. "Atopy" reflects questionnaire-based self-report and was not necessarily physician-diagnosed.

Group	Condition	Cohort n (%)	Population benchmark (context only)
**Neurodevelopmental / Neurological**	ADHD (Attention Deficit Hyperactivity Disorder)	4 (5.4%)	~4.4 % (diagnosed cases, DE)
	Dyslexia	2 (2.7%)	1.9–2.6 % strict, up to 7–15 % broadly defined
	Migraine	4 (5.4%)	~10 % (children/adolescents)
	Other (each n=1; 1.4%)	2 (2.7%)	variable / referral-based (see note)
**Gastrointestinal**	Lactose Malabsorption	7 (9.5%)	~20–40% in clinical groups, lower overall
**Metabolic / Endocrine**	Obesity	2 (2.7%)	5.9 % (obesity), 15.4 % including overweight
	Other (each n=1; 1.4%)	2 (2.7%)	study-dependent (see note)
**Cardiac**	Other (each n=1; 1.4%)	2 (2.7%)	study-dependent (see note)
**Respiratory–Allergic**	Bronchial Asthma	3 (4.1%)	3.5–4 % (12-month prevalence)
	Self-reported atopy (questionnaire-based)	27 (36%)	≥16 % with ≥1 atopic disease
**Other / rare conditions**	Other (each n=1; 1.4%)	9 (12.2%)	variable / often no robust prevalence (see note)

Group	Condition	Cohort n (%)	Population benchmark (context only)
**Overall**	Cystic Fibrosis	0 (0%)	1:3,300–1:4,800 newborns in Germany
	No Prior Illness	37 (50.0%)	n/a

Notes (what is included in “Other (each n=1; 1.4%)”)

- **Neurodevelopmental / Neurological (2× n=1):** Sensory processing disorder; psychogenic gait disorder.
- **Metabolic / Endocrine (2× n=1):** NAFLD; Hashimoto’s thyroiditis.
- **Cardiac (3× n=1):** Coronary fistula; bicuspid aortic valve; mild mitral regurgitation.
- **Other / rare (6× n=1):** Gilbert’s syndrome (Gilbert–Meulengracht syndrome); FASD; Loeys–Dietz syndrome; scoliosis; acne; visual disorders (amblyopia); divergent strabismus; myofascial pain syndrome; chromosome 13 anomaly (Trisomy 13).
