# Supplementary material for: Immune-metabolic trajectories delineate subgroups in paediatric long COVID
Source: Nat Commun. 2026 May 4;17:4023. doi: 10.1038/s41467-026-72224-y (PMC13139442; doi:10.1038/s41467-026-72224-y)
Supplement: Supplementary file 3 — Reporting Summary [file 41467_2026_72224_MOESM3_ESM.pdf]

nature portfolio

Corresponding author(s): Monika Brunner-Weinzierl

Last updated by author(s): 1st of April 2026

Reporting Summary

Nature Portfolio wishes to improve the reproducibility of the work that we publish. This form provides structure for consistency and transparency in reporting. For further information on Nature Portfolio policies, see our Editorial Policies and the Editorial Policy Checklist.

Statistics

For all statistical analyses, confirm that the following items are present in the figure legend, table legend, main text, or Methods section.

n/a	Confirmed	
☐	☒	The exact sample size (n) for each experimental group/condition, given as a discrete number and unit of measurement
☐	☒	A statement on whether measurements were taken from distinct samples or whether the same sample was measured repeatedly
☐	☒	The statistical test(s) used AND whether they are one- or two-sided<br>*Only common tests should be described solely by name; describe more complex techniques in the Methods section.*
☐	☒	A description of all covariates tested
☐	☒	A description of any assumptions or corrections, such as tests of normality and adjustment for multiple comparisons
☐	☒	A full description of the statistical parameters including central tendency (e.g. means) or other basic estimates (e.g. regression coefficient) AND variation (e.g. standard deviation) or associated estimates of uncertainty (e.g. confidence intervals)
☐	☒	For null hypothesis testing, the test statistic (e.g. F, t, r) with confidence intervals, effect sizes, degrees of freedom and P value noted<br>*Give P values as exact values whenever suitable.*
☒	☐	For Bayesian analysis, information on the choice of priors and Markov chain Monte Carlo settings
☒	☐	For hierarchical and complex designs, identification of the appropriate level for tests and full reporting of outcomes
☐	☒	Estimates of effect sizes (e.g. Cohen's d, Pearson's r), indicating how they were calculated

Our web collection on statistics for biologists contains articles on many of the points above.

Software and code

Policy information about availability of computer code

Data collection	Not applicable.
Data analysis	Not applicable.

For manuscripts utilizing custom algorithms or software that are central to the research but not yet described in published literature, software must be made available to editors and reviewers. We strongly encourage code deposition in a community repository (e.g. GitHub). See the Nature Portfolio guidelines for submitting code & software for further information.

Data

Policy information about availability of data

All manuscripts must include a data availability statement. This statement should provide the following information, where applicable:

- Accession codes, unique identifiers, or web links for publicly available datasets
- A description of any restrictions on data availability
- For clinical datasets or third party data, please ensure that the statement adheres to our policy

Data are available from the LongCOVID Consortium upon reasonable request and subject to consortium approval and applicable data protection regulations.

Research involving human participants, their data, or biological material

Policy information about studies with human participants or human data. See also policy information about sex, gender (identity/presentation), and sexual orientation and race, ethnicity and racism.

Reporting on sex and gender	yes
Reporting on race, ethnicity, or other socially relevant groupings	no
Population characteristics	no
Recruitment	yes
Ethics oversight	yes

Note that full information on the approval of the study protocol must also be provided in the manuscript.

Field-specific reporting

Please select the one below that is the best fit for your research. If you are not sure, read the appropriate sections before making your selection.

[x] Life sciences [ ] Behavioural & social sciences [ ] Ecological, evolutionary & environmental sciences

For a reference copy of the document with all sections, see nature.com/documents/nr-reporting-summary-flat.pdf

Life sciences study design

All studies must disclose on these points even when the disclosure is negative.

Sample size	Long COVID cohort: 148 longitudinal observations (n = 74 participants, two visits each). Controls: n = 27 participants, one visit eaach.
Data exclusions	Of 106 evaluated, 78 eligible; 1 excluded (symptoms clearly pre-dated SARS-CoV-2); final LC analysis n=74 with two visits; assay LOD handling as specified.
Replication	No external replication cohort; robustness via two-visit repeated-measures design (LMM) and prespecified sensitivity/technical replicates where applicable. MMLvalidated via bootstrap resampling (1,000 iterations).
Randomization	Not applicable. This was an observational, non-interventional study with no random allocation of participants to groups.
Blinding	NfL testing was performed blinded to clinical/paraclinical data and outcome measures. No additional blinding procedures were specified.

Behavioural & social sciences study design

All studies must disclose on these points even when the disclosure is negative.

Study description	Observational paediatric long COVID cohort study with longitudinal follow-up (two visits) and clinical plus patient-reported assessments.
Research sample	148 observations (n=74 participants; two visits each) and n=27 controls (single time point).
Sampling strategy	Clinic-based cohort; participants enrolled based on predefined eligibility criteria; controls recruited as comparison group.
Data collection	Standardized clinical assessment and questionnaires/scores; blood sampling for immune–metabolic profiling.
Timing	Two study visits per LC participant (longitudinal); controls assessed once.
Data exclusions	Of 106 evaluated, 78 eligible; 1 excluded (symptoms clearly pre-dated SARS-CoV-2); final LC analysis n=74 with two visits.
Non-participation	Not systematically recorded / not applicable beyond screening failures (state as applicable).
Randomization	Not applicable (non-interventional observational study).

Ecological, evolutionary & environmental sciences study design

All studies must disclose on these points even when the disclosure is negative.

Study description	Not applicable (biomedical/clinical cohort study; no ecological/evolutionary/environmental design).
Research sample	Not applicable.
Sampling strategy	Not applicable.
Data collection	Not applicable.
Timing and spatial scale	Not applicable.
Data exclusions	Not applicable.
Reproducibility	Not applicable.
Randomization	Not applicable.
Blinding	Not applicable.

Did the study involve field work? ☐ Yes ☒ No

Field work, collection and transport

Field conditions	Not applicable (no field work; clinical sampling).
Location	Not applicable.
Access & import/export	Not applicable.
Disturbance	Not applicable.

Reporting for specific materials, systems and methods

We require information from authors about some types of materials, experimental systems and methods used in many studies. Here, indicate whether each material, system or method listed is relevant to your study. If you are not sure if a list item applies to your research, read the appropriate section before selecting a response.

Materials & experimental systems

n/a	Involved in the study	
☐	☒	Antibodies
☒	☐	Eukaryotic cell lines
☒	☐	Palaeontology and archaeology
☒	☐	Animals and other organisms
☐	☒	Clinical data
☒	☐	Dual use research of concern
☒	☐	Plants

Methods

n/a	Involved in the study	
☒	☐	ChIP-seq
☐	☒	Flow cytometry
☒	☐	MRI-based neuroimaging

Antibodies

Antibodies used	*Describe all antibodies used in the study; as applicable, provide supplier name, catalog number, clone name, and lot number.*
Validation	*Describe the validation of each primary antibody for the species and application, noting any validation statements on the manufacturer's website, relevant citations, antibody profiles in online databases, or data provided in the manuscript.*

Eukaryotic cell lines

Policy information about cell lines and Sex and Gender in Research

Cell line source(s)	Not applicable.
Authentication	Not applicable.
Mycoplasma contamination	Not applicable.
Commonly misidentified lines (See ICLAC register)	Not applicable.

Palaeontology and Archaeology

Specimen provenance	Not applicable.
Specimen deposition	Not applicable.
Dating methods	Not applicable.

☐ Tick this box to confirm that the raw and calibrated dates are available in the paper or in Supplementary Information.

Ethics oversight	Not applicable (human subjects ethics reported under Clinical data).

Note that full information on the approval of the study protocol must also be provided in the manuscript.

Animals and other research organisms

Policy information about studies involving animals; ARRIVE guidelines recommended for reporting animal research, and Sex and Gender in Research

Laboratory animals	Not applicable.
Wild animals	Not applicable.
Reporting on sex	Not applicable.
Field-collected samples	Not applicable.
Ethics oversight	Not applicable (human subjects ethics reported under Clinical data).

Note that full information on the approval of the study protocol must also be provided in the manuscript.

Clinical data

Policy information about clinical studies

All manuscripts should comply with the ICMJE guidelines for publication of clinical research and a completed CONSORT checklist must be included with all submissions.

Clinical trial registration	Not applicable (observational, non-interventional cohort study; not a clinical trial).
Study protocol	Protocol approved by the local ethics committee; written informed consent/assent obtained from participants and/or legal guardians (details provided in Methods).
Data collection	Standardized clinical assessment and patient-reported symptom/functional measures at two visits for LC participants; controls assessed once; biospecimens collected using predefined SOPs.
Outcomes	Primary outcomes were group differences in immune–metabolic and autoantibody/NfL readouts and their associations with clinical symptom/functional measures over time; prespecified analyses using mixed-effects models.

Dual use research of concern

Policy information about dual use research of concern

Hazards

Could the accidental, deliberate or reckless misuse of agents or technologies generated in the work, or the application of information presented in the manuscript, pose a threat to:

No	Yes	
x		Public health
x		National security
x		Crops and/or livestock
x		Ecosystems
x		Any other significant area

Experiments of concern

Does the work involve any of these experiments of concern:

No	Yes	
x		Demonstrate how to render a vaccine ineffective
x		Confer resistance to therapeutically useful antibiotics or antiviral agents
x		Enhance the virulence of a pathogen or render a nonpathogen virulent
x		Increase transmissibility of a pathogen
x		Alter the host range of a pathogen
x		Enable evasion of diagnostic/detection modalities
x		Enable the weaponization of a biological agent or toxin
x		Any other potentially harmful combination of experiments and agents

Plants

Seed stocks	Not applicable.
Novel plant genotypes	Not applicable.
Authentication	Not applicable.

ChIP-seq

Data deposition

- [ ] Confirm that both raw and final processed data have been deposited in a public database such as GEO.
- [ ] Confirm that you have deposited or provided access to graph files (e.g. BED files) for the called peaks.

Data access links<br>*May remain private before publication.*	Not applicable.
Files in database submission	Not applicable.
Genome browser session<br>(e.g. UCSC)	Not applicable.

Methodology

Replicates	Not applicable.
Sequencing depth	Not applicable.
Antibodies	Not applicable.
Peak calling parameters	Not applicable.
Data quality	Not applicable.
Software	Not applicable.

Flow Cytometry

Plots

Confirm that:

- [x] The axis labels state the marker and fluorochrome used (e.g. CD4-FITC).
- [x] The axis scales are clearly visible. Include numbers along axes only for bottom left plot of group (a 'group' is an analysis of identical markers).
- [x] All plots are contour plots with outliers or pseudocolor plots.
- [x] A numerical value for number of cells or percentage (with statistics) is provided.

Methodology

Sample preparation	Serum/plasma analysed using a bead-based multiplex cytokine assay run on a flow cytometer; samples processed according to the manufacturer’s protocol; standard curves included on each run/plate and provided in the Supplementary Information.
Instrument	Flow cytometer used for bead acquisition: LSRFortessa.
Software	LEGENDplex Data Analysis Software
Cell population abundance	Not applicable (bead-based cytokine quantification); results reported as concentrations (e.g., pg/mL) derived from standard curves.
Gating strategy	Not applicable; bead-based assay; concentrations reported.

- [x] Tick this box to confirm that a figure exemplifying the gating strategy is provided in the Supplementary Information.

Magnetic resonance imaging

Experimental design

Design type	Not applicable.
Design specifications	Not applicable.
Behavioral performance measures	Not applicable.

Acquisition

Imaging type(s)	Not applicable.
Field strength	Not applicable.
Sequence & imaging parameters	Not applicable.
Area of acquisition	Not applicable.

Diffusion MRI ☐ Used ☒ Not used

Preprocessing

Preprocessing software	Not applicable.
Normalization	Not applicable.
Normalization template	Not applicable.
Noise and artifact removal	Not applicable.
Volume censoring	Not applicable.

Statistical modeling & inference

Model type and settings	Not applicable.
Effect(s) tested	Not applicable.

Specify type of analysis: ☐ Whole brain ☐ ROI-based ☐ Both

Statistic type for inference<br>(See Eklund et al. 2016)	Not applicable.
Correction	Not applicable.

Models & analysis

n/a	Involved in the study
☒	☐ Functional and/or effective connectivity
☒	☐ Graph analysis
☒	☐ Multivariate modeling or predictive analysis

Functional and/or effective connectivity	Not applicable.
Graph analysis	Not applicable.
Multivariate modeling and predictive analysis	Not applicable.
